# A fractal pattern of hierarchical genetic population structure in mixed stocks across fish segregated by dams revealed by genomic resources for curimba *Prochilodus lineatus*

**DOI:** 10.1111/jfb.70278

**Published:** 2025-11-23

**Authors:** Gabriel M. Yazbeck, Gésica Aparecida Santana Nascimento, Leiliane Campos Carvalho, Raíssa Cristina Dias Graciano, Rosiane de Paula Santos, Rafael Sachetto Oliveira

**Affiliations:** ^1^ Programa de Pós Graduação em Ecologia Universidade Federal de São João Del Rei São João Brazil; ^2^ Programa de Pós Graduação em Biotecnologia Universidade Federal de São João Del Rei Divinópolis Brazil; ^3^ Laboratório de Recursos Genéticos (LARGE), DEZOO Universidade Federal de São João Del Rei São João Brazil; ^4^ Departamento de Ciência da Computação Universidade Federal de São João Del Rei São João Brazil

**Keywords:** genomics, microsatellites, migratory fish, Neotropics, population genetics

## Abstract

Genomic resources, new microsatellite markers and a novel observation of a fractal pattern in the genetic population structure are presented for curimba, *Prochilodus lineatus*, a freshwater migratory model species of South America. Our main goals were to investigate the presence of mixed fish stocks and the effects of damming‐induced fragmentation, as well as hatchery releases, over the genetic diversity of this species population. We focused on the upper segment of the highly fragmented Grande River, Minas Gerais, Brazil, within the megadiverse La Plata River basin. Using maximum likelihood estimation, different genetically admixed co‐occurring fish stocks were identified. These served as input for a second analysis, the results of which were further examined as a hierarchical genetic structure investigation. Each of these three iterative steps consistently revealed two newly rearranged internal groups within the previously defined clusters (2^3^). We suggest that this nested pattern of mixed stocks in the main river channel reflects distinct, well‐defined breeding sites that are differentially distributed across the basin's spatial scale levels (main river, first‐order tributaries, second‐order tributaries and so on). These potential sites would be geographically arranged based on a fractal disposition, mirroring the self‐similar organization of the bottom‐up stream network. This would imply homing behaviour in *P. lineatus*. Our work also provides high‐throughput molecular data, including the first scaffold‐level draft genome assembly for this species, alongside a valuable set of microsatellites for rapid, low‐cost DNA marker development. The findings will aid researchers and environmental managers in addressing conservation challenges of Neotropical migratory fishes. The results support the hypothesis of isolating effects caused by dams and suggest restocking influences local genetic variability. Importantly, the study highlights the role of tributaries of different orders in harbouring genetically diverse populations of migratory freshwater fishes. We also introduce the concept of *quasi*‐demes, defined as nearly random‐mating population units measurable at arbitrary scales across the basin's landscape, which become increasingly similar to true demes when smaller tributaries are considered.

## 1 INTRODUCTION

The curimba *Prochilodus lineatus* Valenciennes 1836 (Characiformes: Prochilodontidae), also known as sabálo, grumatá, streaked prochilod and other common names, is one of the most important freshwater migratory species for inland fisheries in South America. It corresponds to >50% of the fish biomass from the La Plata basin (Bowen, 2022a, 2022b), a megadiverse hydrological system (Cassemiro et al., 2023), reaching Argentina, Bolivia, Brazil, Paraguay and Uruguay. This major Neotropical basin is heavily impacted by anthropogenic activity (Reis et al., 2016). *P. lineatus* also naturally occurs in the Paraíba do Sul basin (Brazil) and has been recorded as an invasive species in China (Endruweit, 2014) and Vietnam (Kalous et al., 2012).

*P. lineatus* has a conspicuous specialized inverted funnel mouth, an adaptation from its detritivorous habits (Bowen, 2022b), and, due to its abundance and popularity, it is an emblematic example of the Neotropical potamodromous (i.e., freshwater migratory) life history. Reproductive migrations of these fishes are triggered by environmental cues, such as increasing daylight, the onset of the tropical rains and the lunar phase (e.g., Lopes et al., 2018). These upstream runs are regionally denominated *piracema*, a term used to refer both to the migratory process and to the fish species which partake in it. It typically occurs from around the early austral summer through to the end of the season (Carolsfeld et al., 2003). Thus, this process is potentially sensible to ongoing climate change (Alix et al., 2020).

*Piracema* fishes naturally spawn upon reaching upstream breeding grounds (e.g., Ribolli et al., 2020). Larvae and juveniles then passively drift downstream and reach nursery and feeding floodplains, where they grow and mature (Petry et al., 2003). Adults spawn several consecutive years during their life cycle (Carolsfeld et al., 2003). Although *piracema* fishes include species from other taxonomic orders, they are composed primarily of Characiformes and Siluriformes.

Species from the *Prochilodus* genus were among the first Neotropical migratory fishes to have its ex situ spawning mastered. This was motivated by the intensive exploitation of hydroelectricity generation and its impact over migratory species, aiming restocking operations, intended to counteract dwindling fisheries around regions affected by dams (Saraiva & Pompeu, 2016).

These restocking practices have been largely abandoned in some relevant places for hydroelectric production, such as the Brazilian state of Minas Gerais (MG), in the past decade. This was, in part, due to general relaxation of environmental protection‐oriented regulations (Zellhuber, 2016), although a rigorous, holistic (i.e., ecological, genetic and anthropological) assessment of the activity's potential benefits or pitfalls is still lacking. Given its status as the main targeted fish for stock enhancement in the past 50 years or so, *P. lineatus* is a natural candidate as a model species for gauging the impacts of dams and the effectiveness of possible mitigation measures regarding *piracema* fishes.

Fishes under the impacts of hydroelectric power production may benefit from objective genetic information raised through molecular markers such as microsatellites, which are amenable to simple genotyping routines for hatchery or wild fish monitoring operations (Wenne, 2023). The popularization of next‐generation sequencing (NGS) techniques has made the characterization of new DNA markers increasingly accessible and straightforward (Yazbeck et al., 2024). Genomic resources currently available for *Prochilodus* species include full mitochondrial DNA sequences (Carmo et al., 2016; Chagas et al., 2016; Santos et al., 2021), NGS data for *P. lineatus* (Stornioli et al., 2021) and a reference genome for *Prochilodus magdalenae* (Yepes‐Blandón et al., 2022).

Mixed fish stocks can follow a genetically *mixed* population model, characterized by each individual's genome originating from a single ancestral population source (genetic cluster), out of a *K* number of alternatives, where these demes do not share breeding grounds and events. Alternatively, mixed fish stocks might be portrayed by an *admixture* model. It considers different individual proportions of genomic heritage out of several ancestral genetic clusters where previous generations have potentially engaged in common reproductive events (Pritchard et al., 2000; Wang, 2022). Admixture, thus, provides a convenient framework for the problem of unravelling the genetic structure of *piracema* fishes under the impact of dams and decades of stocking operations largely conducted without planned considerations towards genetic issues.

Prior to the use of microsatellite and admixture methods, Sivasundar et al. (2001) detected no genetic population structure in *P. lineatus*, attributing this to its strong migratory ability. Rueda et al. (2013) were the first to apply both methods, revealing seasonal stock segregation in the Lower Uruguay River and suggesting mixed stocks for fisheries management. Later, Perini et al. (2021) described complex dynamics between the Pardo and Lower Grande rivers. Ferreira et al. (2017) investigated *P. lineatus* stocks from the Middle and Upper Paraná basin, whereas Rosa et al. (2022) studied those from the Mogi‐Guaçu River, detecting temporal genetic structuring. Ferreira et al. (2023) also reported this along with mixed stocks in the Paranapanema River basin. These investigations point to the importance of free‐flowing tributaries for spawning in this species, reporting high genetic variability and generally resolving two or three genetic admixture clusters. No genetic structure analysis exists for *P. lineatus* from the Upper Grande River, the uppermost region of the La Plata River basin's eastern flank.

The Grande River springs from an altitude of almost 2,000 m in Minas Gerais and runs through 1360 km until its confluence with the Paranaíba River, where both coalesce onto the Paraná River (a major component of the La Plata basin). It is an illustrative example of thoroughly impounded river, with 12 large dams, with the Camargos hydroelectric power plant being the last dam upbound implemented around 1960. It is closely followed downstream by another dam from the Itutinga power plant, operational since 1955, which delimits a singular, relatively small closed impoundment (without tributaries).

Immediately downstream the Camargos dam, a conservation‐oriented hatchery was active for decades until 2019 in broodstock management and fish stocking operations of a largely unaccounted number of fry, fingerlings and adult fishes (possibly dozens of millions), mainly of *P. lineatus*. These stocking operations primarily occurred throughout tributaries from the Upper Grande River basin (particularly around municipalities upstream the Camargos dam). More relevant to this study, 9000 fry and fingerlings from this species were released in situ in 2016 at the enclosure between the Camargos and Itutinga dams, besides the likely occurrence of unintentional escapes from adult fish, often kept at net‐pens in the reservoir. It provides a singularly convenient opportunity for evaluating the local genetic effects of restocking.

Further downstream lies the relatively new Funil power plant dam (constructed around the year 2000), the only dam accommodating a fish transposition mechanism, a lift (Suzuki et al., 2011). The degree in which these large dams genetically isolate *P. lineatus* in the Upper Grande River is not currently known nor are the genetic effects from fish stocking there.

Our core objectives in this work were (i) testing the hypothesis of the occurrence of mixed stocks across *P. lineatus* populations segregated by the past three upbound dams from the Upper Grande River, MG, Brazil; (ii) testing the hypothesis that dams are genetically isolating migratory fish in the region; and (iii) testing the hypothesis that fish stocking practices have altered genetic diversity in populations of fish trapped between dams. We aimed at doing so through the development of a genome‐wide set of microsatellite *loci* for rapid marker development in *P. lineatus* while making available new genomic resources for this fish, a model for Neotropical freshwater migratory species in general.

## 2 MATERIALS AND METHODS

### 2.1 Ethics statement

All procedures presented in this study were conducted under the guidance and authorization of the Committee on Ethics in Animal Use (CEUA‐UFSJ) under licences 27/2011 and 6991130223, as well as CGEN licences A9D0E5 and AC5A784, and SISBIO licences 37222‐2 and 85497‐1. No experimental assays were performed on live specimens.

### 2.2 Genomics, bioinformatics and microsatellite marker development

**FIGURE 1:**
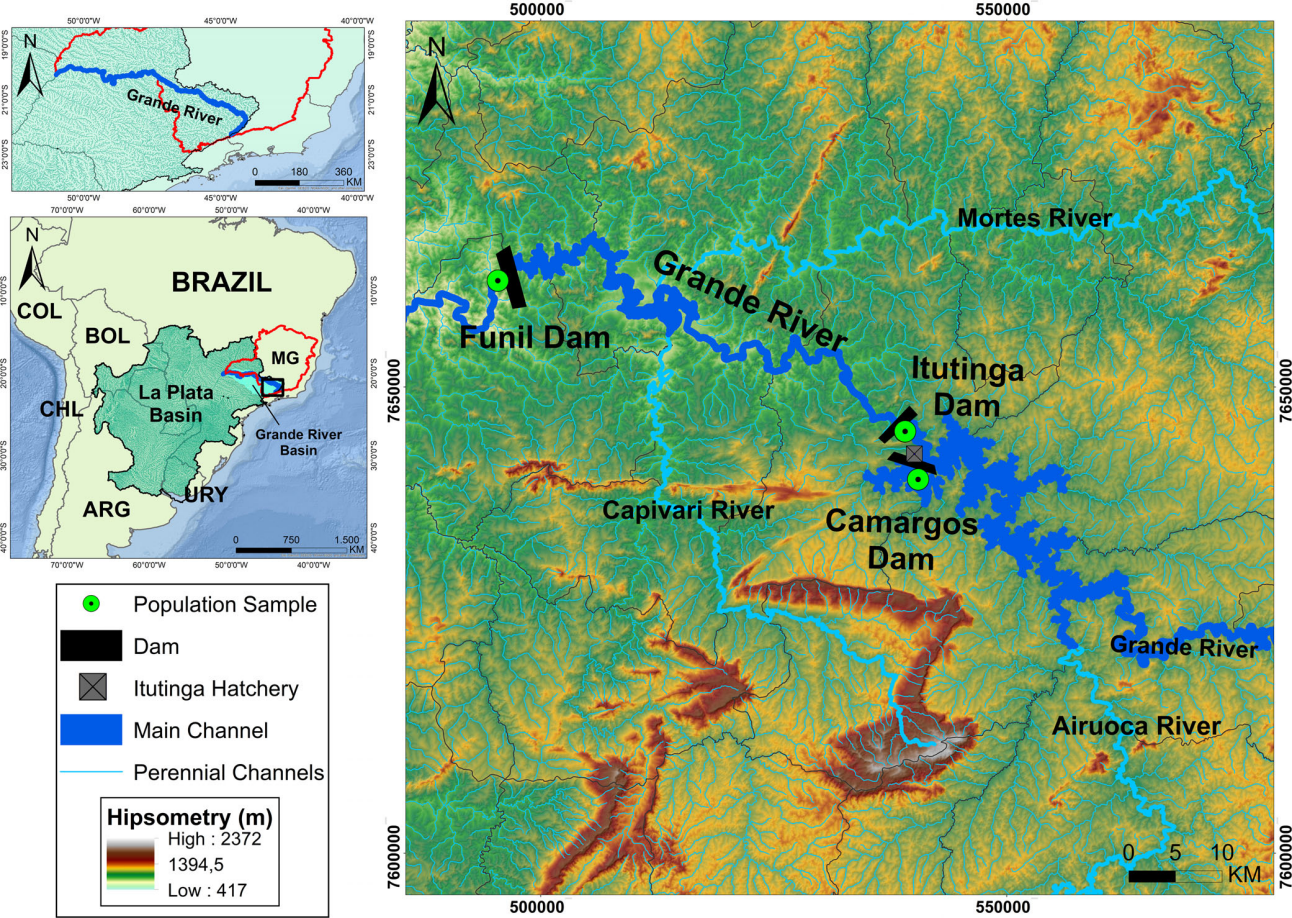
Geographical location of the studied population samples for curimba *Prochilodus lineatus* from the Upper Grande River basin, MG, Brazil.

Genomic libraries of whole‐genome shotgun fragments (500 bp) were produced for NGS out of a single adult female individual from the Itutinga Hatchery Station (21°19′20.7″ S 44°36′56.9″ W, Figure 1). The methodological steps and parameters for NGS and initial microsatellite characterization were the same as presented in Yazbeck et al. (2018) and carried out by an external service provider (SP) BGI (Tai Po, Hong Kong). The resulting 90 bases paired‐end (PE) short‐reads were organized as three sets of twin FASTQ files. We verified the quality of DNA short‐reads using MultiQC (Ewels et al., 2016) and computed all of its 21‐mers (k‐mer substrings) in jellyfish (Marçais & Kingsford, 2011). These results were used to estimate the expected genome size of *P. lineatus* through a frequency histogram in R (version 4.4.1, R Core Team, 2024).

We used all the DNA short‐reads, along a set of fasta format contigs containing microsatellites resolved by the SP upon the original undisclosed assembly, to execute a de novo genomic assembly. For this step, we used MEGAHIT (Li et al., 2015), using k‐mer = 47, to generate new contigs and, then, submitting these to the SOAPdenovo‐fusion module from SOAPdenovo 2 (Luo et al., 2012) to resolve scaffolds. This genome assembly was processed in the *Sagarana* high‐performance computing cluster (CEPAD‐ICB‐UFMG, Belo Horizonte, Brazil). The National Center for Biotechnology Information's (NCBI – Sayers et al., 2022) contamination screening tool FCS (Astashyn et al., 2024) was used for filtering out mitochondrial assembly, sequences from microorganisms and library adaptors found in the final scaffold‐level sequence. Assembly statistics were accessed using assemblyStats (http://gif.biotech.iastate.edu) and visualized with assembly‐stats 17.02 (Challis, 2017).

We mapped the microsatellites characterized in the steps above in the new genomic assembly following the strategy delineated in Yazbeck et al. (2018). A sequence alignment map (SAM) was then produced using BBmap (Bushnell, 2014) by mapping short‐reads unto the genomic assembly and finally converted to a binary format (BAM) using SAMtools (Li et al., 2009).

For this work, we developed new species‐specific microsatellite markers to be applied in the population genetics analysis. We used the results of the genomic microsatellite *loci* search by the SP for choosing candidate *loci* for empirical tests. The microsatellites were sorted by decreasing motif size and number of repeats to maximize chances of finding polymorphic *loci* (Brandström & Ellegren, 2008). A total of 22 candidate primer pairs were chosen (12 from the top‐sorted candidates and 10 at random) and then synthesized by an SP (Integration DNA Technologies, Coralville, USA). These were applied to wet bench assays in four individual DNA samples. Polymerase chain reaction (PCR) was carried out in a final volume of 10 μL, with initial conditions of 1 μL of unquantified Chelex‐extracted genomic DNA to seed the reaction, 10 pmol of each primer, MgCl_2_ (2 mM), Tris–HCl (10 mM), KCl (50 mM), 0.2 mM of dNTP and 1 unit of *Taq* DNA polymerase enzyme (Phoneutria, Belo Horizonte, Brazil). The thermal PCR cycles were as follows: initial genomic DNA denaturation at 94°C for 4 min, followed by 30 iterated cycles of denaturation at 94°C for 30 s, primers annealing temperature (specific for each primer pair) for 30 s and amplicon elongation step at 72°C for 30 s, with a final elongation step at the same temperature for 5 min.

The resulting PCR products were visualized using 10% polyacrylamide gel electrophoresis at ~15 V/cm, with a 25‐bp DNA fragment size ladder stained with ethidium bromide. *Loci* yielding polymorphic patterns were then assayed in a sample of 20 individuals to optimize PCR conditions. Gels were photographed under UVB light and scored using GelAnalyzer. Scored DNA fragments were then converted to nominal allele classes using an automated binning approach under supervision based on frequency histograms and formatted for downstream analysis. Band scoring was cross‐validated by at least two authors.

### 2.3 Genetic diversity

A total of 100 *P. lineatus* individual tissue samples (ethanol‐preserved fin fragments) were used from the DNA bank of the *Laboratório de Recursos Genéticos* (LARGE‐UFSJ). These samples were collected in 2013 from three locations (Figure 1) in the Upper the Grande River, MG: downstream the Funil dam, upstream the Itutinga dam and upstream the Camargos dam (see Suzuki et al., 2011 for a more detailed characterization from this general encompassed area). An additional sample of 40 individuals was obtained from the Itutinga dam reservoir in 2023 (Table 1).

**TABLE 1:** Evaluated *Prochilodus lineatus* individuals sampled from the last three dams from the Upper Grande River, MG, Brazil.

Sample	ID	Location	Year	*N*
Camargos	1–20	21°20′27.1″S 44°36′34.3″W	2013	20
Itutinga 1	21–60	21°17′39.9″S 44°37′24.1″W	2013	40
Itutinga 2	61–100	21°17′30.4″S 44°37′11.4″W	2023	40
Funil	101–140	21°08′39.4″S 45°02′19.1″W	2013	40
Total				140

Abbreviations: ID, individual label; *N*, sample size.

After DNA marker development experiments, a total of 15 new empirically validated microsatellite *loci* were applied to 140 individuals of *P. lineatus*. Hardy–Weinberg equilibrium (HWE) and linkage disequilibrium (LD) were tested for each population sample using the methods implemented in GenePop 007 (Rousset, 2008), with default options for Markov‐chain Monte‐Carlo (MCMC) parameters. These were analysed under new significance threshold values for multiple tests following sequential Bonferroni corrections. Microchecker (van Oosterhout et al., 2004) was used for controlling scoring errors (stuttering, large allele dropout and null alleles). Genetic data handling, conversion and visualizations were mainly conducted with the adegenet package for R (Jombart, 2008). Polymorphic information content (PIC) was calculated according to Botstein et al. (1980).

Genetic diversity estimates for the population samples were obtained using GenAlex (Peakall & Smouse, 2012). We calculated expected and observed heterozygosities (*H*_E_ and *H*_O_, respectively) and allelic richness, here expressed as the number of alleles (*N*_A_), to assess diversity within population samples. Jackknife estimates of *N*_A_ were conducted using EstimateS (Colwell & Elsensohn, 2014), and confidence intervals (CIs) were calculated in R. Rarefaction analysis of *N*_A_ was performed to control for sample size bias by retaining 20 samples, using the dplyr, tidyr and pegas packages in R (Paradis, 2010). Visualizations were plotted using ggplot2.

### 2.4 Population structure

Jost's differentiation statistic D, which focuses on allelic diversity among samples, rather than heterozygosity, was computed for its suitability for markers with multiple alleles. We also applied Hedricks's unbiased and standardized fixation index G“_ST_. It is devised for circumventing small and differential sample sizes of genotypes from distinct demes or locations (Meirmans & Hedrick, 2011) and normalized by the maximum theoretical differentiation possible under observed homozygosity levels (Hedrick, 2005). Therefore, it is equally adequate and insightful for hypervariable DNA markers.

A two‐level analysis of molecular variance (AMOVA) provided estimates of the variance component structure (φ_ST_), within and among population samples, from pair‐wise Euclidean distances among genotypes. All estimated statistics had its significance levels accessed through 999 random permutations. For D and G”_ST_, standard errors (SE) were calculated by jackknifing over *loci*, and 95% CI were obtained by bootstrapping over *loci* (Peakall & Smouse, 2012).

The genotypic data were also processed using analytical tools implemented in PopCluster (Wang, 2022). A preliminary maximum likelihood estimation (MLE) analysis was performed upon a null‐model for admixture (i.e., a strict mixture model, where an individual composite genotype is assigned exclusively to a single ancestral deme) for clustering individuals into *K* demes. It employs an annealing algorithm, which avoids estimates from being confined within a suboptimal peak, enhancing its chances of finding the global maximum value from the data. These results were, then, used as a departure point for performing MLE of individual admixture coefficients. This allows for the refinement of the assignment of individuals to one of the *K* possible population clusters without supervision (i.e., information from the sample's origin). Unequal allele frequencies were assumed, and the weak scaling factor was used. The optimal *K* value explaining sample substructure (*K* = 1–14, *N* = 100 replicate runs) was primarily based on the second‐order rate of log‐likelihood change, with the *D_LK2_* estimator, together with computations of the F_STIS_ estimator (Wang, 2022). This initial procedure was labelled as a first‐order analysis of population genetic structure.

Then, to explore possible hierarchically hidden strata of genetic structure (Janes et al., 2017; Vähä et al., 2007), the best replicate run results for the chosen *K* value were used for rearranging samples into new clusters. Each of these was, in turn, independently subjected to a second‐order admixture analysis, ranging from *K* = 1 to *K* = 7. This was reiterated once more as a third‐order analysis of population structure, assaying *K* = 1–4. The resulting rearranged clusters across hierarchical orders were examined for genetic diversity and structure measurements as the original population samples.

We also performed a parallel Bayesian inference using the STRUCTURE software suite (Pritchard et al., 2000) under the admixture model, conducted de novo (i.e., without population sample information). Separate Dirichlet hyperparameters (*α*) were estimated for each inferred cluster, allowing flexible prior distributions. Additionally, the analysis assumed correlated allele frequencies across clusters. A total of 5 × 10^5^ steps were discarded before computing 1 × 10^6^ MCMC iterations, with *K* varying from 1 to 8 and 20 replicates each. The best *K* value for the STRUCTURE analysis was primarily estimated using the methods implemented in Kfinder (Wang, 2019).

## 3 RESULTS

### 3.1 Genomics, bioinformatics and microsatellite marker development

Approximately 483.7 million short‐reads (43.53 gigabases) were generated for *P. lineatus* and deposited at the Sequence Read Archive (SRA) database from NCBI (Bioproject accession code PRJNA1120875). Its CG content was 42%, and per base quality value (Q) ranged from 31 to 39, with an average of 38 per read. Based on the k‐mer counts, total genome size was estimated at 1.65 Gb.

**FIGURE 2:**
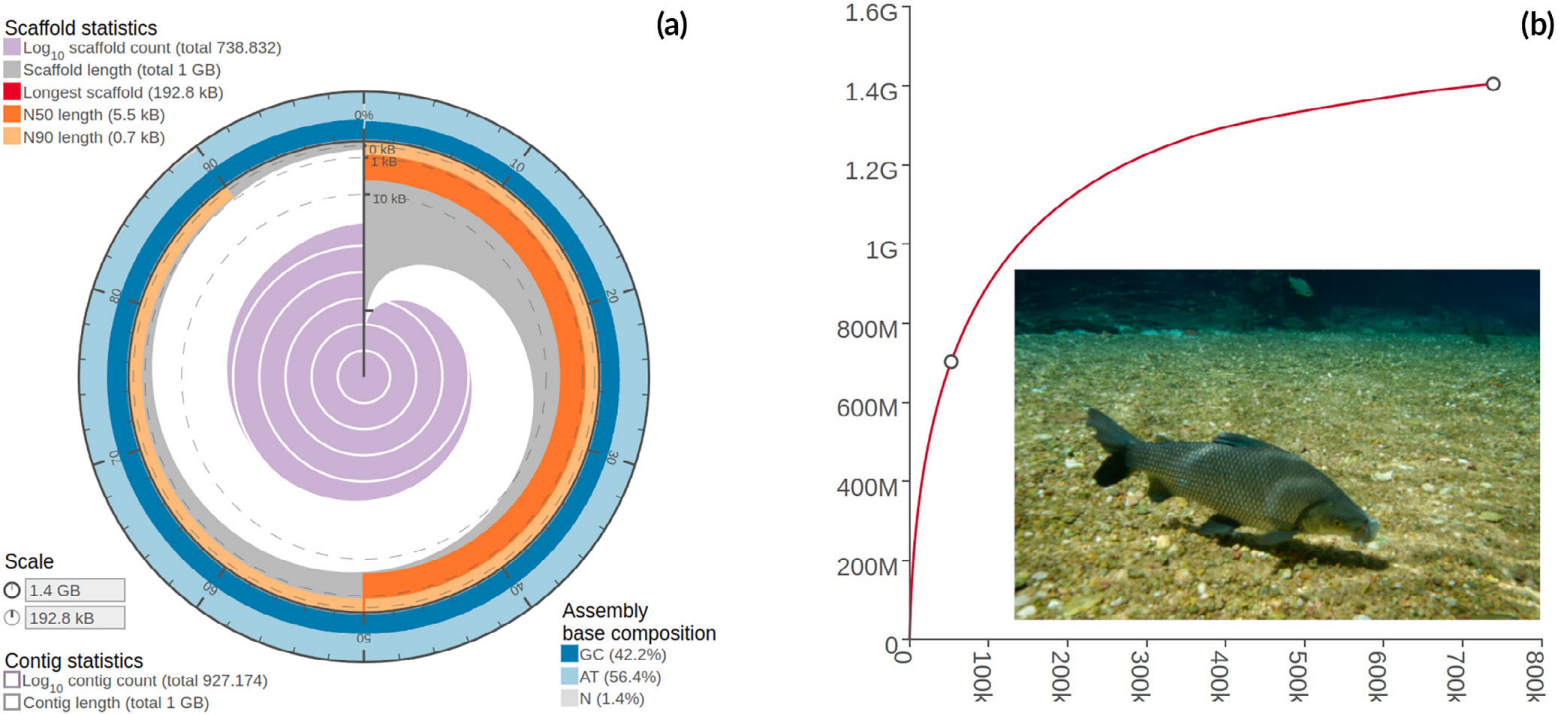
Genome assembly statistical features of *Prochilodus lineatus*. (a) The radius inside the circular plot marks the length of the longest scaffold resolved, and the grey segments represent the cumulative percentage of the assembly contained in scaffolds equal or longer than a particular length. (b) Cumulative contig/scaffold length of the genomic assembly as a function of the number of sequences resolved. The point in the middle of the curve marks the L_50_ feature, the smallest number of sequences, which the sum surpasses half the total genome assembly length (point at the end of the curve − photography by André Seale). G = gigabases and k = thousands of sequences.

The de novo genome assembly generated was 1.4 Gb, with ~31X coverage. This Whole Genome Shotgun project has been deposited at DDBJ/ENA/GenBank under the accession JBGFTF000000000. The version described in this paper is JBGFTF010000000. The total number of contigs/scaffolds was 738,832 sequences, with N_50_ = 5503 bp and L_50_ = 52,610. The average length of contigs was ~1901, with the longest being 192,837 bp (Figure 2). The BAM file associated with it can also be retrieved from NCBI (PRJNA1120875).

A total of 37,476 perfect di‐ to hexa‐nucleotide microsatellite *loci* for *P. lineatus* were characterized in silco and compiled as supporting information (Supplementary Files 1 and 2). These were mapped to the genome assembly, but around 12% of putative *loci* could not be traced back to it. This set comprises approximately 50% di‐, 13% tri‐, 28% tetra‐, 6% penta‐ and 3% hexa‐nucleotides.

Of the 22 primer pairs tested, five (Prol14, Prol15, Prol18, Prol31 and Prol52) resulted in complex amplification patterns and two (Prol56 and Prol28) failed to amplify. Fifteen new microsatellite markers were successfully amplified, and the results for the pooled sample of 140 fish are presented in Table 2. Three markers required adjustments from the originally tested PCR conditions: Prol02 and Prol19 used ~6.7 pmol of each primer, with the latter using 35 thermal cycles, and Prol54 also used 35 cycles. The average number of alleles per *locus* was 22.7 (±1.3 SE). The PIC values were above 0.9 for all *loci*, except Prol53 (PIC = 0.683), considering the whole sample.

**TABLE 2:** Newly validated microsatellite markers for *Prochilodus lineatus*, along with the observed amplified DNA fragment length intervals for each *locus* and their respective defined allelic classes.

*Locus*	Primer sequence	Motif	*T*_m_ (°C)	Size range (bp)	*N*_A_	*N*
Prol01	F: CAAAAGAAAAACGAAAATAAAAGAAACA	AGAT	54.0	109–261	24	117
	R: CCCTCTGGGATCAATATAGTATGTATGT					
Prol02	F: TTTAAACTCATTCAACCGGCCTAC	TATC	54.0	113–389	24	119
	R: GTTGGAGGTTACTTGATAGGGAGGTA					
Prol03	F: TTCCCCATCTTTAGAAATGTCCTTC	AGAT	63.6	134–375	27	122
	R: CTCCTGGGGATAAACCATCAATCT					
Prol04	F: TCTGAAACTATTTTTATAGGTAGGGGGA	TAA	56.4	113–264	25	125
	R: TCCTCTCACCTACAAACTTCCTCAA					
Prol05	F: TCTATCCATCCAATCTATCCATCCA	ATCT	58.8	89–276	31	114
	R: AAATTCCATAGTTTGGAGTGACGC					
Prol06	F: GGCCACTATAAAAGGTAGGCACAA	AGAT	54.0	121–227	17	104
	R: CATACGCTGTCCTAAATGTTCAGC					
Prol07	F: TGGAGGCTGCAGGTATAAGTTTTT	TTA	56.4	102–205	20	94
	R: AAGGCTGGTTCCCTAACCTCC					
Prol08	F: AAGGGCAAGATCAGACAAACAAG	TAT	63.6	146–288	16	122
	R: GAATACTACGCTCATTTCAGAAGAAAAA					
Prol10	F: CACAGACAGACACATATGGGGA	AT	61.2	145–316	25	100
	R: CAGGTATGTTCTATTGAAATGCTAACAC					
Prol11	F: AGAGCTTCAATAGGCTGAAGTCGT	TAGA	56.4	113–222	23	110
	R: TGAATTTCCCTCTGGGATCAATAA					
Prol12	F: GTTGCACCTTTGTAGAGGGGTATG	TCA	56.4	117–280	23	107
	R: ACAGCCGTGGATATAGTCAGAAGC					
Prol19	F: TAAAATGTGGTTTCCTGTGCAATG	TAGA	60.0	87–200	18	106
	R: TTTGCATTTTATCCATCTTTTCCC					
Prol53	F: ATTGCCTGTGTCACTTAAGCATGT	AGAT	51.0	127–209	13	137
	R: TGCTACTGGCTGCTAAATTTCCTT					
Prol54	F: CGAGACGTCTAGCTGACACCATAA	AGAT	49.0	96–309	28	110
	R: TTGACTTCTTGCTGTCATGTCAAA					
Prol57	F: AATGAAACACAGTCAGGCTCCTTC	ATA	49.0	139–317	27	122
	R: TTCCTTCAGGCAATGCACTTTTAT					

Abbreviations: F, forward primer; *N*, sample size (successfully genotyped specimens); *N*_A_, number of alleles; R, reverse primer; *T*_m_, primers annealing temperature.

The amplification/genotyping failure rate varied from 2.1% (Prol53) to 32.8% (Prol07), with an average of 18.6% (±7.9 SE), including instances where one allele was scored and the other deemed undetermined for the same diploid genotype. Six DNA samples (four from Camargos, one from Itutinga 1 and one from Funil) failed to amplify between 5 and 10 *loci*. These were removed from the dataset, and the most downstream analyses were performed with and without them in parallel. Marginal or no differences were observed when comparing results and, thus, these samples were retained.

### 3.2 Genetic diversity

Adjusted significance for 60 multiple HWE tests was set to *p* < 0.008 following a sequential Bonferroni correction. By this criterion, only six *loci* were deemed in HWE. The only *locus* found to conform to expected HWE proportions within each and every population sample, Prol07, was nevertheless not in equilibrium across all four pooled population samples (*p* = 0.002). All deviations were towards heterozygote deficits. Tests for LD within population samples had an adjusted significance level ɑ = 0.0001. No LD was inferred, except for six instances: Prol06 and Prol54 for Camargos; Prol01 and Prol19, Prol05 and Prol57 for Itutinga 1; Prol01 and Prol06, Prol04 and Prol07 for Itutinga 2; and Prol53 and Prol57 for Funil. The following *loci* pairs exhibited LD for the pooled sample of fish: Prol01 and Prol06; Prol04 and Prol07; Prol01 and Prol19; Prol06 and Prol54; Prol05 and Prol57; and Prol53 and Prol57. There was no evidence of scoring errors due to stuttering or large‐allele dropout; however, the presence of null alleles was suggested as a possibility due to the general excess of homozygotes for most allele size classes, for all *loci*, in all population samples, except for Prol07.

Allelic richness ranged from 10.8 (±0.8 SE) alleles for Camargos to 16.9 (±0.9 SE) alleles for Itutinga 2 (Table 3). This trend was confirmed by the jackknife estimates of allelic richness (Supplementary Figure 1), along with the results for the rarefaction analysis (Supplementary Figure 2). Camargos also had the lowest expected heterozygosity, while displaying the highest inbreeding coefficient value, along with the Itutinga 2 sample (Table 3). All population samples exhibited private alleles, with 7 alleles for Camargos and 37 alleles for Itutinga 2 (average 19.7 ± 12.7 SE).

**TABLE 3:** Genetic diversity results for each microsatellite maker across population samples in *Prochilodus lineatus*.

Sample	*Locus*	*N*	*N*_A_	*H*_O_	*H*_E_	*F*	HWE (*p*)
Camargos	Prol01	17	14	0.65	0.91	0.29	0.0023
	Prol02	15	14	0.60	0.89	0.33	0
	Prol03	13	13	0.31	0.91	0.66	0
	Prol04	17	10	0.41	0.87	0.53	0
	Prol05	14	13	0.36	0.9	0.6	0
	Prol06	14	9	0.43	0.81	0.47	0
	Prol07	15	10	0.73	0.85	0.14	0.0191^ns^
	Prol08	13	4	0.08	0.65	0.88	0
	Prol10	16	10	0.69	0.85	0.19	0.0049
	Prol11	17	12	0.35	0.88	0.6	0
	Prol12	13	6	0	0.77	1	0
	Prol19	12	9	0.50	0.84	0.41	0.0004
	Prol53	18	9	0.33	0.77	0.57	0
	Prol54	17	15	0.41	0.91	0.55	0
	Prol57	17	11	0.41	0.87	0.53	0
	Over *loci*	15.2	10.6	0.42	0.85	0.52	0
Itutinga 1	Prol01	33	16	0.27	0.91	0.7	0
	Prol02	33	18	0.55	0.92	0.41	0
	Prol03	31	16	0.52	0.91	0.43	0
	Prol04	36	13	0.31	0.9	0.66	0
	Prol05	26	22	0.62	0.94	0.34	0
	Prol06	34	11	0.21	0.87	0.76	0
	Prol07	20	12	0.95	0.88	−0.08	0.0157^ns^
	Prol08	38	10	0.45	0.85	0.48	0
	Prol10	33	20	0.73	0.94	0.22	0
	Prol11	30	16	0.57	0.89	0.37	0
	Prol12	31	13	0.16	0.9	0.82	0
	Prol19	30	13	0.23	0.91	0.74	0
	Prol53	39	9	0.31	0.64	0.52	0
	Prol54	34	20	0.38	0.92	0.59	0
	Prol57	38	16	0.47	0.91	0.48	0
	Over *loci*	32.4	15	0.45	0.89	0.50	0
Itutinga 2	Prol01	34	19	0.29	0.92	0.68	0
	Prol02	36	18	0.56	0.91	0.39	0
	Prol03	39	19	0.36	0.93	0.61	0
	Prol04	33	14	0.27	0.91	0.7	0
	Prol05	38	24	0.39	0.94	0.58	0
	Prol06	27	14	0.11	0.89	0.87	0
	Prol07	27	18	0.81	0.91	0.1	0.0498^ns^
	Prol08	39	14	0.62	0.87	0.3	0
	Prol10	27	15	1	0.91	−0.1	0
	Prol11	33	17	0.27	0.88	0.69	0
	Prol12	32	17	0.34	0.91	0.62	0
	Prol19	31	13	0.13	0.89	0.86	0
	Prol53	40	10	0.18	0.65	0.73	0
	Prol54	34	19	0.29	0.92	0.68	0
	Prol57	36	22	0.81	0.92	0.13	0.0210^ns^
	Over *loci*	33.7	16.9	0.43	0.89	0.52	0
Funil	Prol01	33	16	0.15	0.92	0.84	0
	Prol02	35	17	0.49	0.91	0.46	0
	Prol03	39	21	0.51	0.95	0.46	0
	Prol04	39	17	0.51	0.92	0.44	0
	Prol05	36	27	0.56	0.95	0.42	0
	Prol06	29	12	0.21	0.79	0.74	0
	Prol07	32	11	0.91	0.85	−0.07	0.4959^ns^
	Prol08	32	13	0.66	0.86	0.24	0
	Prol10	24	18	1	0.93	−0.08	0.7323^ns^
	Prol11	30	18	0.4	0.92	0.57	0
	Prol12	31	19	0.29	0.9	0.68	0
	Prol19	33	15	0.15	0.91	0.83	0
	Prol53	40	11	0.23	0.68	0.67	0
	Prol54	25	14	0.72	0.91	0.2	0.0052
	Prol57	31	21	0.71	0.93	0.23	0
	Over *loci*	32.6	16.67	0.50	0.89	0.44	0
Overall		140	341	0.45	0.91	0.50	0

*Note:* Probability for Hardy–Weinberg test had significance set at *ɑ* = 0.008. All probability values (*p*) are significant, except where noted (ns), and values deemed zero are actually extremely low non‐zero values <0.00009.

Abbreviations: F, inbreeding coefficient; HWE, exact test for Hardy–Weinberg equilibrium; *N*, sample size; *N*_A_, number of alleles; *H*_O_, observed heterozygosity; *H*_E_, expected heterozygosity.

### 3.3 Population structure

The AMOVA results showed that only 1.6% of total variation was explained by the four population samples, with the remaining 98.4% being explained by variation among individuals (φ_ST_ = 0.016, *p* = 0.001, degrees of freedom = 139). All pair‐wise fixation estimates for G”_ST_ and Jost's D were significantly different from zero, including both samples collected in the same location at different times (Table 4). Itutinga samples (2013 and 2023) had the lowest values, whereas Camargos showed the highest overall pair‐wise variation and differentiation for both the respective indices. Considering all 15 microsatellite *loci*, among the four population samples, the fixation index G”_ST_ reached 0.16 (±0.03 SE; 0 to 0.26 CI, *n* = 999; *p* = 0.001), and the total genetic differentiation estimate of Jost's D was 0.15 (±0.03; 0 to 0.24 CI, *n* = 999; *p* = 0.001).

**TABLE 4:** Summary of pair‐wise fixation index G”_ST_ (below diagonal) and Jost's genetic distance D (above diagonal) for microsatellite diversity among population samples of *Prochilodus lineatus* from the Upper Grande River, MG.

	Camargos	Itutinga 1	Itutinga 2	Funil
Camargos	‐	0.156**	0.222**	0.203**
Itutinga 1	0.171**	‐	0.066*	0.087**
Itutinga 2	0.241**	0.072*	‐	0.167**
Funil	0.221**	0.095**	0.180*	‐

* *p* < 0.05.

** *p* ≤ 0.001.

**FIGURE 3:**
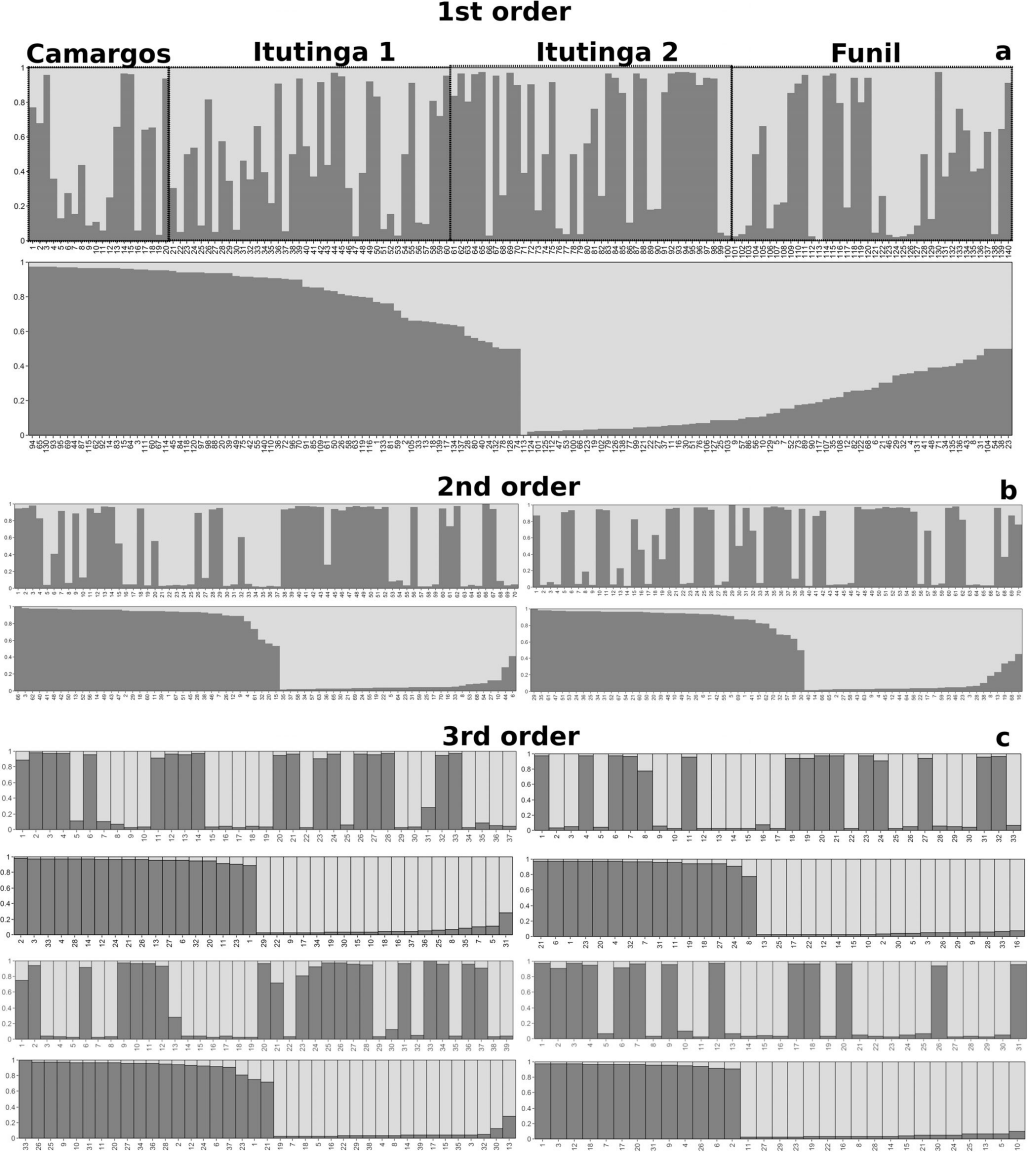
Individual admixture coefficients (bars) for *K* = 2 across inclusive levels of hierarchical genetic structure of *Prochilodus lineatus* populations from the Upper Grande River, MG, Brazil. Different shades of grey represent alternative clusters of origin, with the proportional size of bars indicating the respective genomic contributions from ancestral demes. For each panel, the diagram above shows the sample in the original input sequence, whereas the diagram below shows the sample organized as newly defined clusters. (a) First‐order analysis, showing fish grouped based on the population sample and original ID. (b) Second‐order analysis from clusters defined in the first order. (c) Third‐order analysis of the second‐order clusters. For second and third orders, the numbers below bars are arbitrary and not equivalent to the original ID.

MLE of admixture (Figure 3) pointed to *K* = 2 as the most likely number of clusters, according to Wang's *D*_*LK2*_ metric. However, F_STIS_ suggested *K* = 4 as the most likely number of groups (Supplementary Figure 3). The first‐order admixture analysis allowed the definition of two clusters (with respective individual IDs, Figure 3a). Estimates of *D*_*LK2*_ for both these clusters yielded the value of *K* = 2 as optimal in the second‐order analysis (Figure 3b), whereas F_STIS_ recorded values of *K* = 5 and *K* = 7 (Supplementary Figure 4). Finally, these four newly defined individual rearrangements were studied in the third‐order analysis (Figure 3c), and *D*_*LK2*_ consistently pointed to *K* = 2 for each and every subject cluster (Supplementary Figure 5; individual assignments for all hierarchical orders can be found in Supplementary File 3). We addressed and discarded the possibility of panmixia (*K* = 1) at this and each hierarchical‐order level by directly inspecting log‐likelihood values of the clustering analysis against the results for the assumed value for *K* (Supplementary File 4; Supplementary Table 1).

These different MLE sample arrangements showed a consistent decrease in intrapopulation diversity parameters for the higher orders, such as expected heterozygosity and number of alleles (Supplementary File 4; Supplementary Table 2), whereas interpopulation indicators (G”_ST_ and Jost's D) steadily increased (Supplementary File 4; Supplementary Tables 3–5). The HWE analysis of MLE‐defined clusters indeed showed a tendency towards Hardy–Weinberg expectations in higher‐order group arrangements. In average, third‐order clusters had more *loci* in HWE, with one particular cluster showing eight *loci* in equilibrium (Supplementary File 5).

**FIGURE 4:**
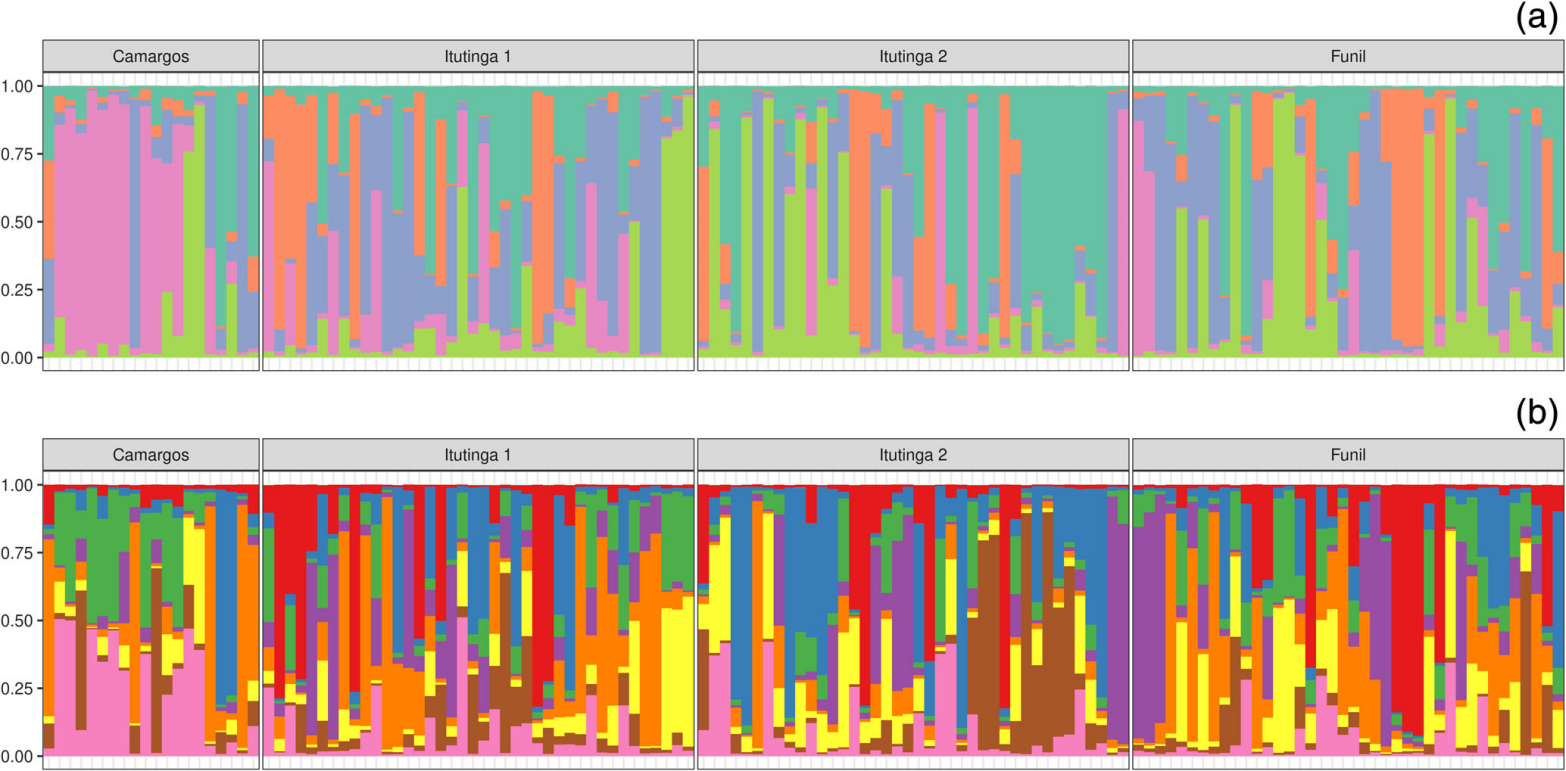
Individual admixture plots from the Bayesian clustering results for (a) *K* = 5 and (b) *K* = 8 of *Prochilodus lineatus* from the Upper Grande River, MG. The order of the samples are the incremental ID numbers, as presented in Table 1. Each colour represents a different cluster. Note the apparent differentiation at Camargos and Itutinga 2.

The Bayesian approach to admixture also resulted in alternative values for best *K* estimates. According to Pritchard's p(X|*K*), the best is *K* = 8, with a log likelihood of −8990.74, indicating a maximum posterior probability of p(*K*|X) ≈ 1 compared to other *K* scenarios. Concurrently, both Evanno's ΔK and Wang's parsimony estimators resolved *K* = 5. Figure 4 presents individual admixture estimates ordered by population samples across the alternative *K* scenarios.

## 4 DISCUSSION

### 4.1 Genomics, bioinformatics and microsatellite marker development

Genome assemblies have become mandatory for many different biological disciplines (Jung et al., 2020). Our work takes a new step towards this goal by publicly presenting the first comprehensive genomic resources for this highly iconic species, *P. lineatus*. The volume of short‐read DNA sequence data presented here surpasses the only previously available FASTQ data for *P. lineatus* (Stornioli et al., 2021) by a factor of 12. This number of sequences is, though, one order of magnitude below the short‐read data reported for the trans‐Andean congeneric species, *P. magdalenae* (Yepes‐Blandón et al., 2022), which, however, is not currently publicly available. The CG content and quality rate of reads were the same as the ones previously presented for other *piracema* fish (e.g., Graciano et al., 2022; Yazbeck et al., 2018). It's also the most extensive dataset of short‐reads publicly disclosed so far for any member of the Prochilodontidae family.

This work provides the first de novo genome assembly for *P. lineatus*. The reported assembly is slightly longer than the genome size described for *P. magdalenae* (Yepes‐Blandón et al., 2022), despite its extensive fragmentation, and shorter than the size estimated by the k‐mer method, which is not always precise (Pflug et al., 2020). The limited length of the short‐reads used here contributed to curtailing our genome assembly, primarily motivated by the exclusive search of microsatellite *loci*. The full sequence of this *P. lineatus* specimen's mitogenome was previously presented in Santos et al. (2021). This novel genome assembly might be explored for the initial annotation of longer‐characterized sequences (e.g., Graciano et al., 2021).

The genome‐wide microsatellite set produced here, with more than 37,000 putative *loci*, can now be free and easily accessed for the rapid, low‐cost development of potentially hundreds of new empirically validated markers. It may also be explored for technological development of practical tools for hatcheries and management of impacted areas, such as multiplexed sets of markers (e.g., Carvalho et al., 2018). Some of these resources may also, arguably, be applied in different fishes, particularly in other prochilodontid and characid species (e.g., Barbosa et al., 2006; Carmo et al., 2015). The relatively high rate of success in empirical assays attests to the high quality of this genome‐wide microsatellite set. These 15 new DNA markers presented here increase the number of available microsatellites for *P*. *lineatus* by ~84%.

We observed a variable rate of missing data among these new markers, with some *loci* (e.g., Prol07) presenting a relatively pronounced amount. The reason for this is likely multifactorial, knowingly, the degree of DNA sample conservation, lack of amplification success or refraining from scoring uncertain banding patterns, such as ‘shadows’ (from extension slippage) and heteroduplexes (cross‐annealing of single‐stranded DNA from alleles with different repeat counts).

One noteworthy possibility for *loci* with variable rate of missing data among samples is that it could have been characterized from a supernumerary (or B) chromosome. *P. lineatus* (2 *N* = 54) have been described with a variable number of up to nine B chromosomes (Stornioli et al., 2021). Indeed, a quick BLAST search in the specimen's genome for the PliSat14 probe (NCBI accession MZ161107) associated with B chromosomes in *P. lineatus* (Stornioli et al., 2021) yields several hits (e.g., contigs C526428, C83258, C1638590 and C591000). Nevertheless, we have not yet associated these to any microsatellite marker used in this study.

Fortunately, approaches such as admixture assignments are found to be reasonably insensible to missing data, especially when it is not due to a systematic cause (Falush et al., 2007; Wang, 2022). Even null alleles might have only a marginal impact (Budowle and Sajantila, 2024; Carlsson, 2008; Putman & Carbone, 2014). For some other applications, the issue of missing data is more critical (e.g., estimating effective population size, *N*_*e*_ – Peel et al., 2013).

The microsatellite Prol53 had a particularly high rate of successful genotype scoring. Despite exhibiting the lowest allelic richness and polymorphic information content, this *locus* had the most weight in explaining the two main clusters from the whole pooled data through a principal component analysis (results not shown).

The number of alleles resolved across the 15 *loci* was analogous to previous reports on the characterization and application of genome‐specific microsatellite markers in this fish (e.g., Rueda et al., 2011; Yazbeck & Kalapothakis, 2007). The set of validated markers are deemed as highly informative as all showed PIC values above the arbitrary 0.5 threshold. Despite Microchecker suggesting the possible presence of null alleles for most *loci* in all populations, we consider this an artefact due to the inherent sample substructuring, which leads to critical confounding effects in this analysis (Waples, 2015).

### 4.2 Genetic diversity

A study by Wang et al. (2021) suggests that applying fewer than 11 or 12 microsatellite markers may lead to important bias in population genetics structure assessments. The results presented here are the most extensive efforts so far in terms of the number of *loci* analysed in *P. lineatus*. It revealed high levels of microsatellite diversity in fish from the uppermost portion of the Grande River, MG, despite the influence of large dams. This result is consistent with previous studies on the population genetics of *P. lineatus* with comparable genomic sampling efforts (e.g., Perini et al., 2021; Rueda et al., 2013).

Despite the smaller sample size from Camargos (addressed through jackknife and rarefaction analysis for the allelic richness) and the variable rate of genotyping, the genetic diversity analysis within‐population samples suggests a lower genetic richness in fish upstream of the Camargos dam. It is the last artificial obstacle towards the headwaters of the Grande River basin. This is an area considered as priority for conservation of migratory fishes in the state of Minas Gerais (Drummond, 2005), and it has been knowingly segregated from the downstream system for the past 65 years since the construction of the Camargos power plant dam. It does not bear a fish passage and larvae, and juveniles are not likely to successfully drift downstream (Suzuki et al., 2011). Hatchery‐based restocking of *P. lineatus* did occur in the region for the past decades (Saraiva & Pompeu, 2016). Yet, prior to more recent scientifically driven actions, fish stocking was mostly executed without purposefully defined goals, other than fulfilling regulatory agencies' licensing demands or in the context of environmental education actions. This portion of the river is still connected to a last major free‐flowing upstream Grande River tributary, the Aiuruoca River, MG (Figure 1).

The Camargos dam likely isolates fish from the downstream hydrographic system, and stocking operations might have been limited in providing adequate gene flow connectivity for the system upstream. Its local populations are most likely undergoing stronger random genetic drift, which leads to genetic impoverishment within demes and differentiation among them. This notion is corroborated by the results showing the largest amount of interpopulation variation and differentiation in Camargos from all other samples. Thus, our results potentially support the hypothesis that dams are isolating fish populations in the Upper Grande River. Other competing possibilities for explaining this observed pattern of genetic variability should be noted as DNA marker artefacts (e.g., faulty primer annealing) or sampling bias (smallest sample size). These issue deserves deeper investigation in future research due to its relevant conservation implications.

The little difference among population samples registered by the AMOVA is compatible with a single, almost panmitic population, or could be explained by more complex, cryptic patterns of mixed genetic variability. Yet, it is deemed as less straightforward in the interpretation of hypervariable markers, such as microsatellites (Jost, 2008). The G”_ST_ fixation index, along with the allelic differentiation estimator Jost's D, pointed to significant deviations from panmixia, with all comparisons significantly different from zero. This supports the hypothesis of isolation by dams over fish unable to freely migrate.

The 2023 sample from the Itutinga reservoir (Itutinga 2) is the most genetically diverse followed by fish caught downstream of the Funil dam. The latter, as first raised in Yazbeck and Kalapothakis (2007), could be preventing migrating fish from downstream, potentially of different stocks, of proceeding further upward, despite its fish lift. This virtual *cul‐de‐sac* could be leading to a higher observed genetic diversity in the sample by accumulation, comparable to the trapped gene pool effect discussed by Perini et al. (2021). Upstream of the Funil dam lie the mouths of two major free‐flowing tributaries of the Grande River, the Mortes River and the Capivari River (Figure 1), which could be natural candidates for harbouring different *P. lineatus* stocks (e.g., Suzuki et al., 2011).

The *P. lineatus* population at Itutinga's reservoir, in turn, has undergone genetic variation and differentiation of around 7% in 10 years. Despite being closed and strongly isolated, it seems to have locally accumulated genetic variation (allelic richness) since 2013. A plausible cause for this could be fish stocking of *P. lineatus* from the hatchery situated on its left bank. Hatchery‐based stocking, or repeated escapes of broodstock from net‐pens in the Camargos reservoir, with varying sources for spawning matrices, along with the marked tendency for genetic bottlenecks during particular reproductive efforts (single or few spawned couples), could be yielding alternative sets of new alleles being introduced into this limited stretch of the Grande River. Although this location had the highest levels of diversity (*N*_*A*_ and *H*_*E*_), it also had the highest inbreeding coefficient, reinforcing the notion of hatchery‐driven substructuring. For more than 5 years now, though, the hatchery station has been operating mainly for scientific research and has ceased its fish stocking in the Grande River, MG, and surrounding region. These results support the hypothesis that hatcheries can influence the genetic diversity in populations of fish trapped between dams.

We conclude that stocking may be effective in preserving genetic diversity in isolated populations from the Upper Grande River basin. However, it should only be carried out after a thorough genetic characterization of existing stocks (see discussion below), with careful consideration of potential local adaptations. Additionally, hatchery breeding strategies must be meticulously planned, and the process should be closely monitored through efficiency evaluations and follow‐up studies.

Regarding our main hypothesis on the occurrence of mixed stocks in the Upper Grande River, MG, the extensive and verified lack of adherence to Hardy–Weinberg proportions in all population samples, always showing a deficit of heterozygotes, along with some verified LD, suggest strong genetic stratification in all sampled populations (i.e., more than one deme being sampled) and, thus, are consistent with its prediction. The fact that it is so pervasive across *loci* allows us to initially disregard other putative confounding factors, such as eventual null alleles. It can be biologically interpreted as an expression of the Wahlund effect, leading to a pronounced downward departure from the expected heterozygosity under HWE.

### 4.3 Population structure

The admixture model seems biologically meaningful and appropriate for studying potamodromous Neotropical fishes. These fish might experience multiple reproductive runs over several years, potentially resulting in variable breeding outcomes with respect to both the moment and place of spawning. More importantly, potential genetically distinct individuals and stocks might end up co‐occurring in the same locality and time during migratory runs or trapped between adjacent dams. This would isolate them from their original breeding grounds, leading to subsequent exogamy among different demes.

The MLE approach for admixture, which estimates population genetics parameters that maximize a likelihood function (Wang, 2003; Wang, 2022), is a powerful and scalable alternative to the more hegemonic Bayesian inference. Bayesian analysis is based on the conditional probabilities of genotypes, given some population genetics parameter, which is, in turn, highly sensible to often subjective prior probabilities (van de Schoot et al., 2021). Because admixture models are highly hyperdimensional (in our particular case, from 326 independent variables, for *K* = 1, up to 6384, for *K* = 14), the MLE path has the advantage of high accuracy, coupled with lower computational demands (Wang, 2022), which allows for more extensive replication. Here, it showed a high degree of convergence across replicates (many have zero variance) for different *K*.

We adopted Wang's *D*_*LK2*_ estimator for determining optimal *K*, as it has been shown to be generally more reliable across a variety of investigated scenarios compared to the F_STIS_ estimator (Wang, 2022). The first‐order MLE analysis of admixture initially pointed for two groups, which did not correlate with sampling scheme. Both clusters contain members from all sampled populations. This strongly suggests the occurrence of mixed stocks of *P. lineatus* from the main channel of the Upper Grande River. Caution was taken to dismiss panmixia as the best possibility by directly examining the log‐likelihood values of alternative models, as *D*_*LK2*_ would not possibly capture *K* = 1 (Wang, 2022), just like Evanno's Δ*K* for Bayesian estimation (Janes et al., 2017), and given that the F_STIS_ estimator would always be zero (Wang, 2022). The results from the Bayesian analysis of admixture also reinforce the notion of several, different fish stocks in our total sample, not correlated with sampling locations or moments, supporting the hypothesis of the occurrence of historical mixed stocks throughout the Upper Grande River basin.

Different values of *K*, thus, can reveal multiple underlying aspects of population structure and distinct stages in the population's history (Gehri et al., 2021; Gilbert, 2016; Janes et al., 2017; Wang, 2022). As discussed by Perini et al. (2021) and Ferreira et al. (2023), mixed stocks might reflect the use of different affluent streams as breeding sites. The two major genetically differentiated groups defined in the first‐order MLE analysis, then, could be the reflex of the existence of two main ancient breeding grounds (e.g., the Mortes, Capivari or the Aiuruoca rivers), defining forks along migratory routes, splitting groups in the Upper Grande River. This result indicates the probable existence of at least two distinct stocks mixed along the studied area, not explained by the recent implementation of dams. Evidence for mixed stocks in *P. lineatus* was also previously forwarded by Rueda et al. (2013) and Avigliano et al. (2019).

Aiming to deepen the exploration of these seemingly co‐occurring genetically mixed stocks of *P. lineatus* from the main channel of the Upper Grande River, the two clusters initially defined by MLE were iteratively unfolded for two more analyses rounds. Consistently, and surprisingly, every examined cluster revealed two newly characterized smaller subgroups, nested within clusters resolved in the immediate previous step (2^3^). Judging by the sorted samples of newly defined clusters from Figure 3c, the F_STIS_ estimates from Supplementary Figure 5 or the HWE analysis from MLE clusters, there would be potentially more admixture degrees to be untangled from further hierarchical analysis, although we would reach rather critical sample sizes. The MLE‐defined clusters showed the expected admixture model behaviour towards higher orders. The third‐order had more genetic homogeneity within clusters and a maximization of genetic variation and distance among clusters. It also showed increasing numbers of *loci* in HWE, attesting to the meaningfulness of the hierarchical admixture analysis and the usefulness of these new microsatellite markers.

The Bayesian exploration of the admixture model suggested deeper levels of substructuring, pointing to as many as eight possible clusters in the whole sample of fish, a scenario compatible with the eight demes defined during the ‘peeling’ of MLE layers. The *K* = 5 scenario was favoured based on methods that balance model fit and parsimony. A fair visual agreement of cluster boundaries can be verified for *K* = 8 when placing the samples accordingly to the clusters defined at *K* = 5 (results not shown). This pattern supports the interpretation of nested clusters within clusters consistent with a hierarchical arrangement of the genetic structure of the mixed stocks.

The observed stratified, self‐similar, hidden pattern of the population genetic structure across the larger sample of fish may reveal the existence of potential different, well‐defined, ancestral breeding grounds, hierarchically nested within differential scale levels, throughout the basin, organized based on fractal geometry (e.g., main river, first‐order, second‐order tributaries and so on). One parsimonious explanatory hypothesis is that these reiterated subdivisions of clusters into two, across scales, reflect a preferential directional navigation tendency, either towards streams on opposite sides or, alternatively, along cardinal orientations, when individuals encounter forks and tributaries (e.g., left‐leaning versus right‐leaning migrators or east versus west, and so forth). This asymmetrical bias would operate at multiple levels within the hydrographic system, partly defining the apparent self‐similar pattern.

This view also helps reconcile the former notion of a single panmitic population exhibiting high levels of genetic variability, alongside low but significant levels of among‐population genetic structure generally reported in this species. One hallmark of a fractal is the capacity to be measured across arbitrary scale levels, with values increasing as higher levels of magnification (i.e., smaller scales) are considered. The observed increase in F_ST_ values at superior hierarchical orders supports this interpretation.

Thus, we propose that populations of *P. lineatus* across the hydrographic landscape may be regarded as *quasi*‐demes, exhibiting almost random mating across arbitrary scale levels throughout its distribution, potentially becoming progressively more similar to true demes as smaller tributaries are examined.

If this association is confirmed, we can conclude that it may constitute the first indirect genetic evidence of a strong degree of homing behaviour in a Characiformes *piracema* fish species. Philopatric spawning, or homing behaviour, a thoroughly investigated issue in salmonid and other migratory species, is still poorly understood in the Neotropics. It has been documented for Neotropical catfishes (e.g., Batista & Alves‐Gomes, 2006; Pereira et al., 2009; Hauser et al., 2020). But most importantly, it was suggested for the congeneric *P. argenteus*, based on radio‐tagging results from the São Francisco River basin (Godinho & Kynard, 2006) and discussed for *P. lineatus* itself by Careaga and Carvajal‐Vallejos (2019), from morphometric data. Our work revives this area of inquiry for migratory fish in South America.

Being particularly abundant and a conspicuously competent, far‐ranging migratory species, we argue that in a pre‐damming past, *P. lineatus* could probably reach the innermost free‐flowing tributaries for spawning, upon the colossal La Plata basin, which cuts through a diversified range of biomes and microhabitats. We hypothesize that this would likely point towards philopatric behaviour as an evolutionary stable strategy due to the possible occurrence of distinct local adaptations, as it has been shown in other fish species (e.g., Mobley et al., 2019; Salles et al., 2016; Vøllestad & Primmer, 2019).

In conservation biology, the understanding of degrees of admixture is important in management decisions (Wang, 2003; Wang, 2022). The confirmation of mixed stocks and the hierarchical genetically stratified nature of *P. lineatus* from the main channel of an important large and intensely dammed river will likely enlighten its future research and fisheries management. If further corroborated, these findings, particularly its ensuing propositions, would have direct implications to environmental and development decision‐making. It highlights the possible central relevance of smaller key tributaries (actively sought for the implementation of new small hydroelectric power plants, e.g., Ferreira et al., 2022) for the conservation of genetic variation in a socio‐environmentally relevant species, as it highlights the importance of free‐flowing tributaries of different orders throughout the basin.

On a broader level, we raise the question whether these apparent fractal layers from the genetic structure could imply in a more widespread phenomena for *piracema*, other migratory fishes or even other non‐aquatic migratory organisms, showing a temporary mixture, but segregated breeding grounds. We believe this is worthy of further investigation, as fractal structures are pervasive in nature, not only in biology (e.g., Bassingthwaighte, 1992; Leggett et al., 2019; Meyer et al., 2020; Sendker et al., 2024), but it is also conspicuously present in major geographical landscape attributes like mountain ranges, forest borders, rivers and coastlines (Andronache et al., 2019; Xu et al., 1993). The only direct discussions on the relationship between fractals and genetic population structure were laid by Wingen et al. (2007), based on computer simulation results of long‐distance dispersal of clonal fungal spores, and by Lee (2020), who explored the fractal dimension of human genetic variability data both within and among genomes.

Our results will very likely benefit researchers and environmental managers in tackling challenging issues related to the conservation of Neotropical migratory fishes, as they support the hypothesis of the isolating effects of dams and local influence of fish stocking over genetic variability. The former seems to have been partially alleviated by the availability of tributaries around affected areas, as previously acknowledged in other *P. lineatus* genetics studies. More importantly, the results presented here point to the potential role of tributaries of different orders in harbouring genetically diverse (possibly unique) populations of migratory freshwater fishes.

## AUTHOR CONTRIBUTIONS

Gabriel M. Yazbeck conceived, conducted and supervised the study, raised funding, performed bioinformatics and population genetics analyses, wrote and edited the manuscript; Gésica Aparecida Santana Nascimento performed marker development and population genetics analysis and wrote the manuscript; Leiliane Campos Carvalho performed marker development; Raíssa Cristina Dias Graciano performed bioinformatics analysis; Rosiane de Paula Santos performed bioinformatics analysis; Rafael Sachetto Oliveira supervised and performed bioinformatics pipelines. All contributors read and agreed upon the final manuscript.

## FUNDING INFORMATION

This study was funded by Conselho Nacional de Pesquisa e Desenvolvimento Tecnológico (CNPq), Coordenadoria de Aperfeiçoamento de Pessoal de Nível Superior (CAPES), Companhia Energética de Minas Gerais (CEMIG), Agência Nacional de Energia Elétrica (ANEEL) and Fundação de Amparo à Pesquisa de Minas Gerais (FAPEMIG).

## Supporting information


**Supplementary File 1.** Microsatellite DNA sequences and primers presented in the plain text format (txt) for *Prochilodus lineatus*. The order of columns is ‘*Locus*’; ‘Original fasta label’; ‘Motif’; ‘Repeats’; ‘5′‐flank’; ‘3′‐flank’; ‘F‐primer’; ‘F‐Tm’; ‘R‐Primer’; ‘R‐Tm’; ‘PCR product’; ‘Product length’; ‘Location’; ‘Scaffold or contig label in the newly presented assembly, when mapped’. This file is available at fisgshare under doi: 10.6084/m9.figshare.26848654. https://doi.org/10.6084/m9.figshare.26848654.
**Supplementary File 2**. List of DNA sequences presented in the fasta format for contigs from the original assembly from where microsatellite *loci* for *Prochilodus lineatus* were originally characterized. This file is available at fisgshare under doi: 10.6084/m9.figshare.26848015 https://doi.org/10.6084/m9.figshare.26848015.
**Supplementary File 3**. *Prochilodus lineatus* population samples, fish IDs and their rearrangements based on the first‐, second‐ and third‐order maximum likelihood estimation (MLE)‐defined clusters. This file is available at fisgshare under doi: 10.6084/m9.figshare.26871031. https://doi.org/10.6084/m9.figshare.26871031.
**Supplementary File 4**. Supplementary tables, including log‐likelihood values of the clustering analysis for panmixia and genetic diversity estimates for *Prochilodus lineatus* maximum likelihood estimation (MLE)‐defined clusters from the first, second and third orders of hierarchical analysis. This file is available at fisgshare under doi: 10.6084/m9.figshare.27020857. https://doi.org/10.6084/m9.figshare.27020857.
**Supplementary File 5**. Hardy–Weinberg exact tests for maximum likelihood estimation (MLE)‐defined clusters of *Prochilodus lineatus* from the first, second and third orders of hierarchical analysis. This file is available at fisgshare under doi: 10.6084/m9.figshare.27020869. https://doi.org/10.6084/m9.figshare.27020869.
**Supplementary Figures 1–5**. Supplementary Figures 1–5 are presented in a single portable document format (pdf) file, with respective captions. This file is available at figshare under doi: 10.6084/m9.figshare.29905916 https://doi.org/10.6084/m9.figshare.29905916.
