# Supplementary material for: A fractal pattern of hierarchical genetic population structure in mixed stocks across fish segregated by dams revealed by genomic resources for curimba Prochilodus lineatus
Source: J Fish Biol. 2025 Nov 23;108(4):1029–46. doi: 10.1111/jfb.70278 (PMC13193354; doi:10.1111/jfb.70278)
Supplement: Supplementary file 1 — Microsatellite DNA sequences and primers presented in the plain text format (txt) for Prochilodus lineatus. The order of columns is ‘Locus’; ‘Original fasta label’; ‘Motif’; ‘Repeats’; ‘5′‐flank’; ‘3′‐flank’; ‘F‐primer’; ‘F‐Tm’; ‘R‐Primer’; ‘R‐Tm’; ‘PCR product’; ‘Product length’; ‘Location’; ‘Scaffold or contig label in the newly presented assembly, when mapped’. This file is available at fisgshare under doi: 10.6084/m9.figshare.26848654. https://doi.org/10.6084/m9.figshare.26848654. Supplementary File 2. List of DNA sequences presented in the fasta format for contigs from the original assembly from where microsatellite loci for Prochilodus lineatus were originally characterized. This file is available at fisgshare under doi: 10.6084/m9.figshare.26848015 https://doi.org/10.6084/m9.figshare.26848015. Supplementary File 3. Prochilodus lineatus population samples, fish IDs and their rearrangements based on the first‐, second‐ and third‐order maximum likelihood estimation (MLE)‐defined clusters. This file is available at fisgshare under doi: 10.6084/m9.figshare.26871031. https://doi.org/10.6084/m9.figshare.26871031. Supplementary File 4. Supplementary tables, including log‐likelihood values of the clustering analysis for panmixia and genetic diversity estimates for Prochilodus lineatus maximum likelihood estimation (MLE)‐defined clusters from the first, second and third orders of hierarchical analysis. This file is available at fisgshare under doi: 10.6084/m9.figshare.27020857. https://doi.org/10.6084/m9.figshare.27020857. Supplementary File 5. Hardy–Weinberg exact tests for maximum likelihood estimation (MLE)‐defined clusters of Prochilodus lineatus from the first, second and third orders of hierarchical analysis. This file is available at fisgshare under doi: 10.6084/m9.figshare.27020869. https://doi.org/10.6084/m9.figshare.27020869. Supplementary Figures 1–5. Supplementary Figures 1–5 are presented in a single portable document format (pdf) fil [file JFB-108-1029-s001.zip › Supplementary Figures 1-5/Supplemental_Figures_REVISED2025AUG.pdf]

List of Supplementary Figures

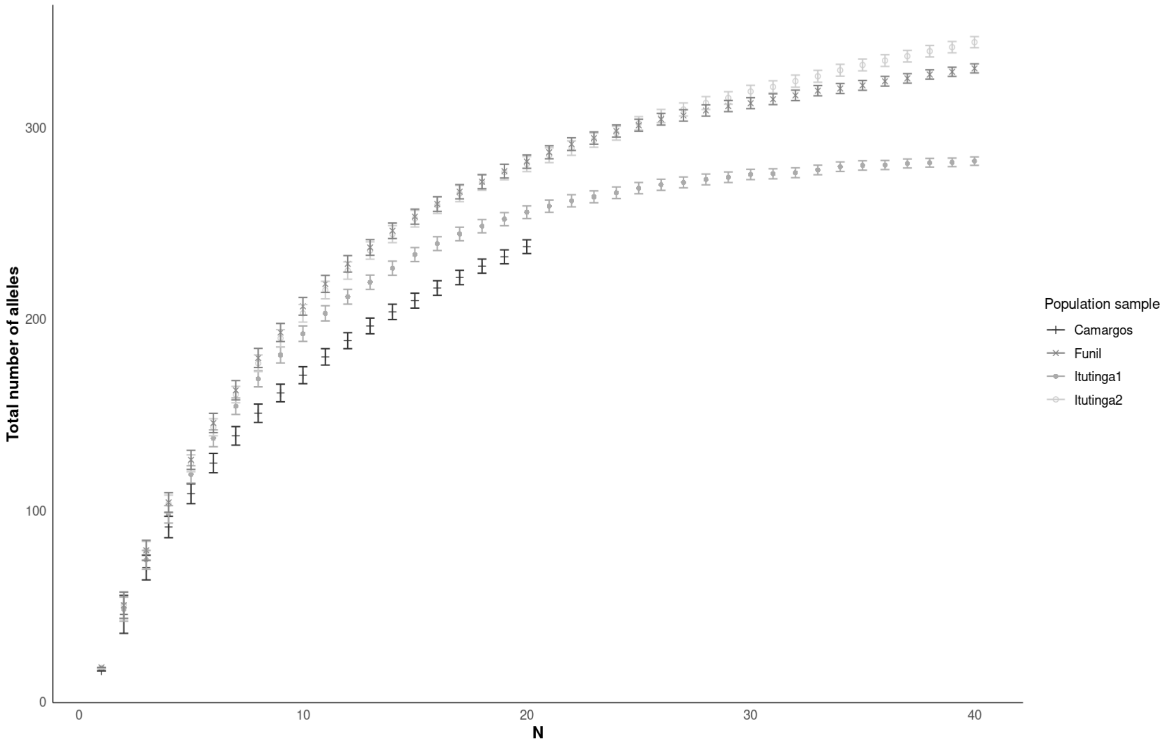

Supplementary Figure 1: Jackknife resampling estimates and 95% confidence intervals (vertical lines) of the total number of alleles (allelic richness), across 341 alleles from 15 *loci* in *Prochilodus lineatus* from four population samples form the Upper Grande, River, MG, Brazil.

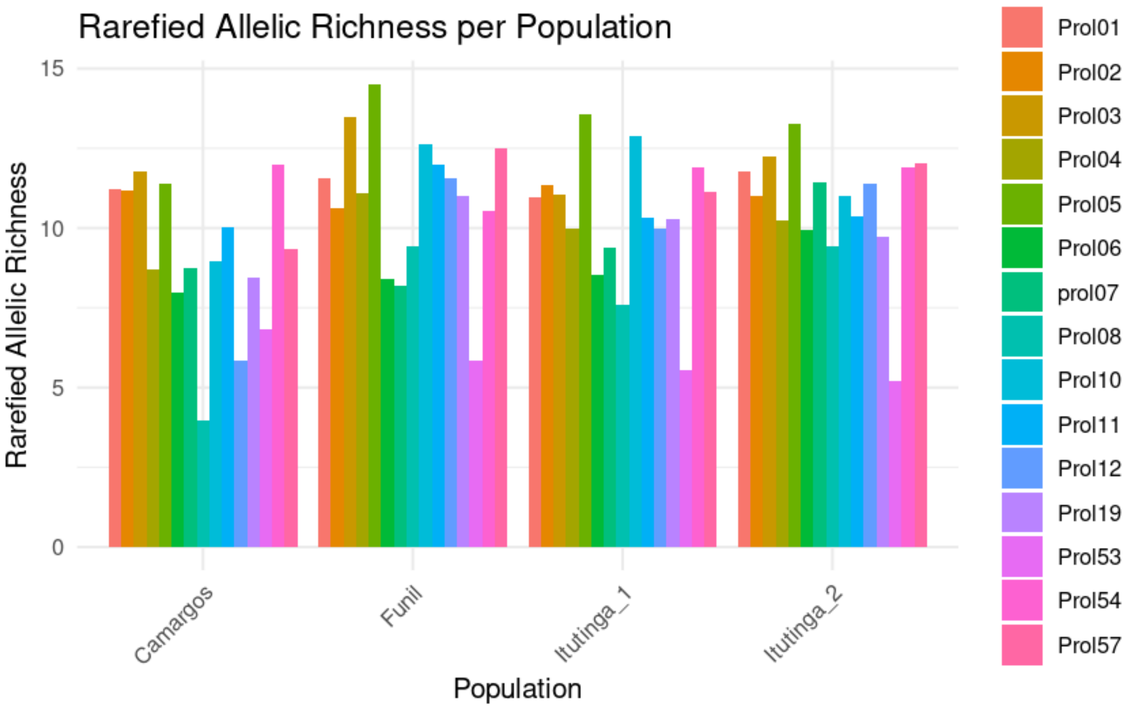

Supplementary Figure 2: Rarefaction estimates of the number of alleles (allelic richness), across 15 *loci* in *Prochilodus lineatus* from four population samples form the Upper Grande, River, MG, Brazil.

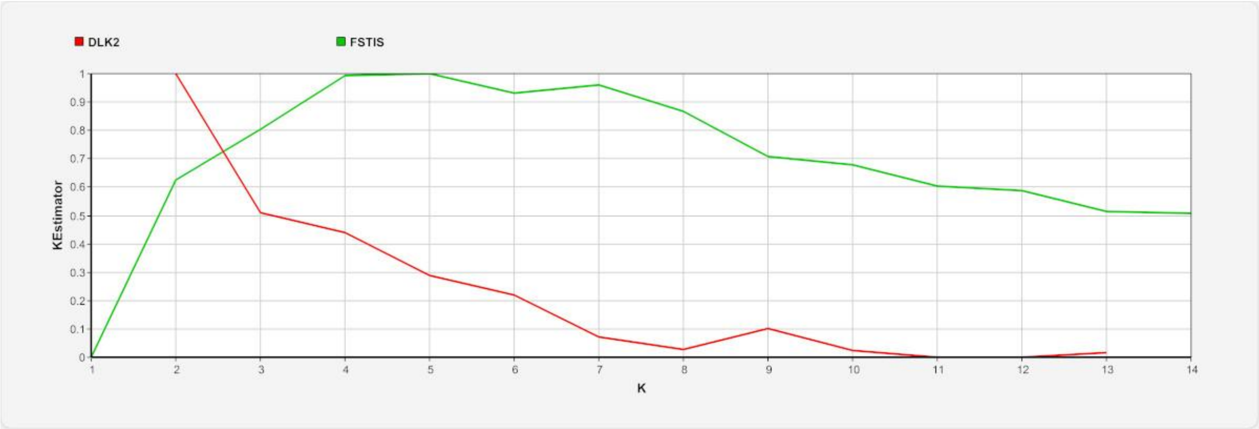

Supplementary Figure 3: Optimal K estimation by two alternative methods, D_{LK2} and F_{STIS} for the 1st order MLE of admixture in *Prochilodus lineatus* from the Upper Grande River, MG.

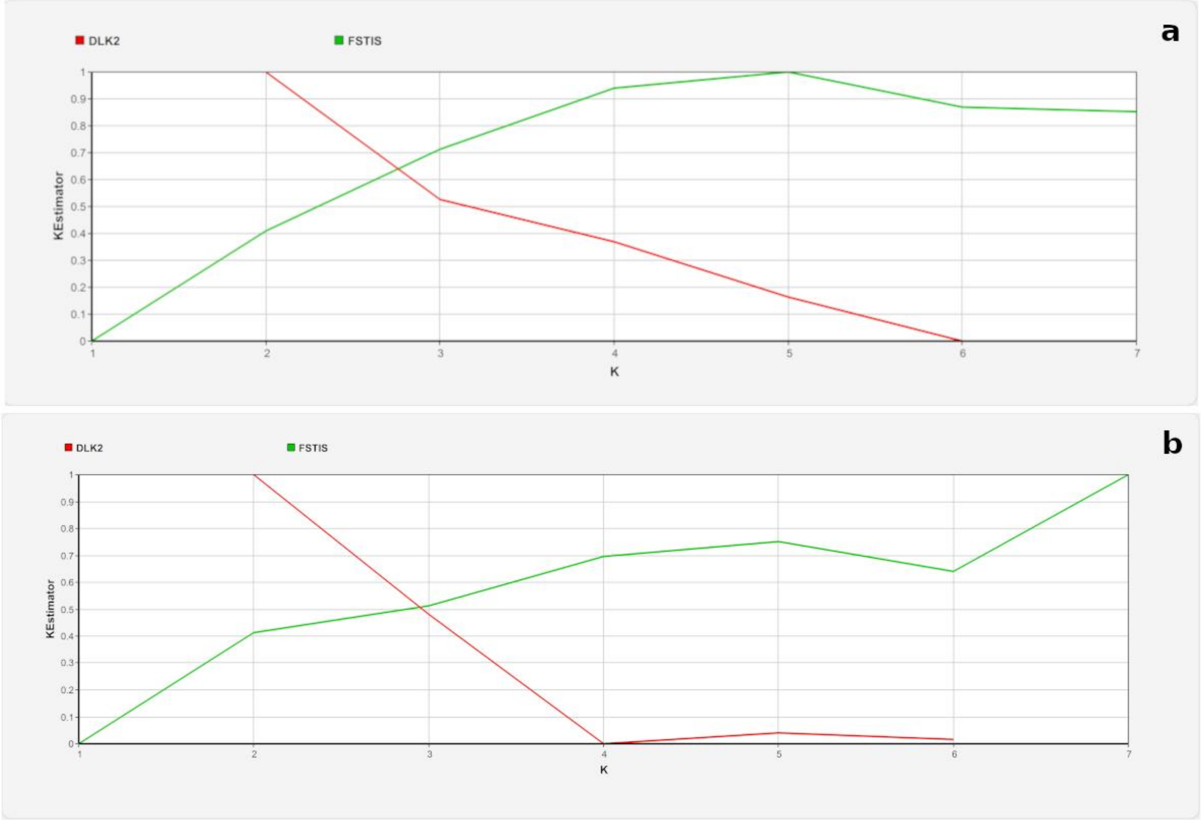

Supplementary Figure 4: Optimal K estimation by two alternative methods, D_{LK2} and F_{STIS} for the 2nd order MLE of admixture in *Prochilodus lineatus* from the Upper Grande River, MG. **a)** Cluster 1 from the 1st order. **b)** Cluster 2 from the 1st order.

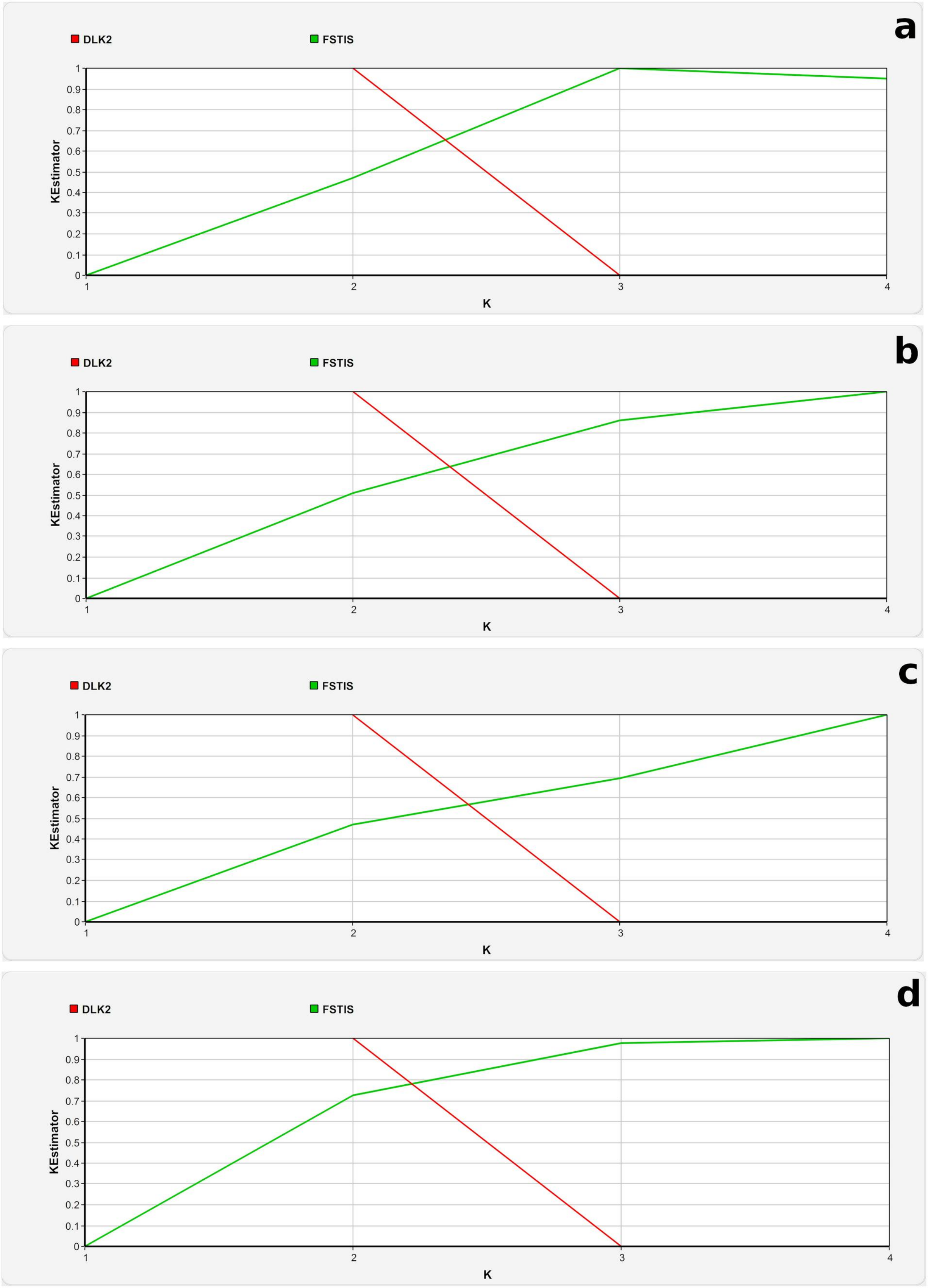

Supplementary Figure 5: Optimal K estimation by two alternative methods, D_{LK2} and F_{STIS} for the 3rd order MLE of admixture in *Prochilodus lineatus* from the Upper Grande River, MG. **a)** Cluster 1 from the 2st order. **b)** Cluster 2 from the 2nd order. **c)** Cluster 3 from the 2nd order. **d)** Cluster 4 from the 2nd order.
