# Supplementary material for: A fractal pattern of hierarchical genetic population structure in mixed stocks across fish segregated by dams revealed by genomic resources for curimba Prochilodus lineatus
Source: J Fish Biol. 2025 Nov 23;108(4):1029–46. doi: 10.1111/jfb.70278 (PMC13193354; doi:10.1111/jfb.70278)
Supplement: Supplementary file 1 — Microsatellite DNA sequences and primers presented in the plain text format (txt) for Prochilodus lineatus. The order of columns is ‘Locus’; ‘Original fasta label’; ‘Motif’; ‘Repeats’; ‘5′‐flank’; ‘3′‐flank’; ‘F‐primer’; ‘F‐Tm’; ‘R‐Primer’; ‘R‐Tm’; ‘PCR product’; ‘Product length’; ‘Location’; ‘Scaffold or contig label in the newly presented assembly, when mapped’. This file is available at fisgshare under doi: 10.6084/m9.figshare.26848654. https://doi.org/10.6084/m9.figshare.26848654. Supplementary File 2. List of DNA sequences presented in the fasta format for contigs from the original assembly from where microsatellite loci for Prochilodus lineatus were originally characterized. This file is available at fisgshare under doi: 10.6084/m9.figshare.26848015 https://doi.org/10.6084/m9.figshare.26848015. Supplementary File 3. Prochilodus lineatus population samples, fish IDs and their rearrangements based on the first‐, second‐ and third‐order maximum likelihood estimation (MLE)‐defined clusters. This file is available at fisgshare under doi: 10.6084/m9.figshare.26871031. https://doi.org/10.6084/m9.figshare.26871031. Supplementary File 4. Supplementary tables, including log‐likelihood values of the clustering analysis for panmixia and genetic diversity estimates for Prochilodus lineatus maximum likelihood estimation (MLE)‐defined clusters from the first, second and third orders of hierarchical analysis. This file is available at fisgshare under doi: 10.6084/m9.figshare.27020857. https://doi.org/10.6084/m9.figshare.27020857. Supplementary File 5. Hardy–Weinberg exact tests for maximum likelihood estimation (MLE)‐defined clusters of Prochilodus lineatus from the first, second and third orders of hierarchical analysis. This file is available at fisgshare under doi: 10.6084/m9.figshare.27020869. https://doi.org/10.6084/m9.figshare.27020869. Supplementary Figures 1–5. Supplementary Figures 1–5 are presented in a single portable document format (pdf) fil [file JFB-108-1029-s001.zip › Supplementary File 3/Supplemental_Table_ID_Cluster_Membership.pdf]

MLE Hierarchical Cluster Membership

		Unsorted		
**ID**	**Sample**	**1st Order**	**2nd Order**	**3rd Order**
1	Camargos	1	1	1
2	Camargos	1	1	1
3	Camargos	1	1	1
4	Camargos	2	3	5
5	Camargos	2	4	7
6	Camargos	2	4	7
7	Camargos	2	4	7
8	Camargos	2	3	5
9	Camargos	2	3	6
10	Camargos	2	4	7
11	Camargos	2	4	8
12	Camargos	2	4	7
13	Camargos	1	1	1
14	Camargos	1	2	3
15	Camargos	1	2	4
16	Camargos	2	3	6
17	Camargos	1	1	2
18	Camargos	1	2	4
19	Camargos	2	3	6
20	Camargos	1	1	1
21	Itutinga_1	2	4	8
22	Itutinga_1	2	4	7
23	Itutinga_1	2	4	7
24	Itutinga_1	1	2	3
25	Itutinga_1	2	3	5
26	Itutinga_1	1	1	2
27	Itutinga_1	2	4	8
28	Itutinga_1	1	1	2
29	Itutinga_1	2	4	8
30	Itutinga_1	2	3	6
31	Itutinga_1	2	4	7
32	Itutinga_1	2	3	6
33	Itutinga_1	1	1	2
34	Itutinga_1	2	3	5
35	Itutinga_1	2	4	8
36	Itutinga_1	1	1	2
37	Itutinga_1	2	4	8
38	Itutinga_1	2	3	5
39	Itutinga_1	1	1	1
40	Itutinga_1	1	2	4
41	Itutinga_1	2	3	5
42	Itutinga_1	1	2	3
43	Itutinga_1	2	3	5
44	Itutinga_1	1	1	1
45	Itutinga_1	1	2	3
46	Itutinga_1	2	4	8
47	Itutinga_1	2	4	8
48	Itutinga_1	2	3	6
49	Itutinga_1	1	1	1
50	Itutinga_1	1	2	3
51	Itutinga_1	2	3	6

MLE Hierarchical Cluster Membership

52	Itutinga_1	2	3	6
53	Itutinga_1	2	3	6
54	Itutinga_1	2	4	7
55	Itutinga_1	1	2	4
56	Itutinga_1	2	3	6
57	Itutinga_1	2	3	6
58	Itutinga_1	1	2	4
59	Itutinga_1	1	2	3
60	Itutinga_1	1	2	4
61	Itutinga_2	1	1	1
62	Itutinga_2	1	2	4
63	Itutinga_2	1	1	2
64	Itutinga_2	1	1	2
65	Itutinga_2	1	2	4
66	Itutinga_2	2	3	6
67	Itutinga_2	1	2	4
68	Itutinga_2	2	3	5
69	Itutinga_2	1	1	2
70	Itutinga_2	1	2	4
71	Itutinga_2	2	4	7
72	Itutinga_2	1	2	4
73	Itutinga_2	2	3	5
74	Itutinga_2	1	2	3
75	Itutinga_2	1	2	3
76	Itutinga_2	2	4	8
77	Itutinga_2	2	3	6
78	Itutinga_2	1	2	3
79	Itutinga_2	2	3	5
80	Itutinga_2	1	1	2
81	Itutinga_2	1	1	2
82	Itutinga_2	2	4	7
83	Itutinga_2	1	1	1
84	Itutinga_2	1	1	1
85	Itutinga_2	1	1	2
86	Itutinga_2	2	4	8
87	Itutinga_2	1	1	1
88	Itutinga_2	1	2	3
89	Itutinga_2	2	4	8
90	Itutinga_2	2	4	8
91	Itutinga_2	1	1	1
92	Itutinga_2	1	1	2
93	Itutinga_2	1	1	1
94	Itutinga_2	1	1	1
95	Itutinga_2	1	1	1
96	Itutinga_2	1	1	2
97	Itutinga_2	1	1	2
98	Itutinga_2	1	1	2
99	Itutinga_2	2	3	5
100	Itutinga_2	2	3	5
101	Funil	2	3	5
102	Funil	2	3	5
103	Funil	2	3	5
104	Funil	2	3	6

105	Funil	1	2	4
106	Funil	2	3	6
107	Funil	2	3	5
108	Funil	2	3	6
109	Funil	1	2	3
110	Funil	1	2	3
111	Funil	1	1	1
112	Funil	2	4	8
113	Funil	2	3	5
114	Funil	1	2	4
115	Funil	1	2	4
116	Funil	1	2	3
117	Funil	2	4	8
118	Funil	1	1	1
119	Funil	1	1	2
120	Funil	1	1	2
121	Funil	2	4	8
122	Funil	2	3	5
123	Funil	2	3	6
124	Funil	2	3	5
125	Funil	2	4	8
126	Funil	2	4	8
127	Funil	2	4	8
128	Funil	1	2	4
129	Funil	2	4	8
130	Funil	1	2	4
131	Funil	2	3	5
132	Funil	1	2	4
133	Funil	1	1	2
134	Funil	1	1	2
135	Funil	2	4	7
136	Funil	2	3	6
137	Funil	1	2	3
138	Funil	2	3	6
139	Funil	1	2	3
140	Funil	1	2	4

Sorted

ID	Sample	1st Order	2nd Order	3rd Order
1	Camargos	1	1	1
2	Camargos	1	1	1
3	Camargos	1	1	1
13	Camargos	1	1	1
20	Camargos	1	1	1
39	Itutinga_1	1	1	1
44	Itutinga_1	1	1	1
49	Itutinga_1	1	1	1
61	Itutinga_2	1	1	1
83	Itutinga_2	1	1	1
84	Itutinga_2	1	1	1
87	Itutinga_2	1	1	1
91	Itutinga_2	1	1	1
93	Itutinga_2	1	1	1
94	Itutinga_2	1	1	1

MLE Hierarchical Cluster Membership

95	Itutinga_2	1	1	1
111	Funil	1	1	1
118	Funil	1	1	1
17	Camargos	1	1	2
26	Itutinga_1	1	1	2
28	Itutinga_1	1	1	2
33	Itutinga_1	1	1	2
36	Itutinga_1	1	1	2
63	Itutinga_2	1	1	2
64	Itutinga_2	1	1	2
69	Itutinga_2	1	1	2
80	Itutinga_2	1	1	2
81	Itutinga_2	1	1	2
85	Itutinga_2	1	1	2
92	Itutinga_2	1	1	2
96	Itutinga_2	1	1	2
97	Itutinga_2	1	1	2
98	Itutinga_2	1	1	2
119	Funil	1	1	2
120	Funil	1	1	2
133	Funil	1	1	2
134	Funil	1	1	2
14	Camargos	1	2	3
24	Itutinga_1	1	2	3
42	Itutinga_1	1	2	3
45	Itutinga_1	1	2	3
50	Itutinga_1	1	2	3
59	Itutinga_1	1	2	3
74	Itutinga_2	1	2	3
75	Itutinga_2	1	2	3
78	Itutinga_2	1	2	3
88	Itutinga_2	1	2	3
109	Funil	1	2	3
110	Funil	1	2	3
116	Funil	1	2	3
137	Funil	1	2	3
139	Funil	1	2	3
15	Camargos	1	2	4
18	Camargos	1	2	4
40	Itutinga_1	1	2	4
55	Itutinga_1	1	2	4
58	Itutinga_1	1	2	4
60	Itutinga_1	1	2	4
62	Itutinga_2	1	2	4
65	Itutinga_2	1	2	4
67	Itutinga_2	1	2	4
70	Itutinga_2	1	2	4
72	Itutinga_2	1	2	4
105	Funil	1	2	4
114	Funil	1	2	4
115	Funil	1	2	4
128	Funil	1	2	4
130	Funil	1	2	4

MLE Hierarchical Cluster Membership

132	Funil	1	2	4
140	Funil	1	2	4
4	Camargos	2	3	5
8	Camargos	2	3	5
25	Itutinga_1	2	3	5
34	Itutinga_1	2	3	5
38	Itutinga_1	2	3	5
41	Itutinga_1	2	3	5
43	Itutinga_1	2	3	5
68	Itutinga_2	2	3	5
73	Itutinga_2	2	3	5
79	Itutinga_2	2	3	5
99	Itutinga_2	2	3	5
100	Itutinga_2	2	3	5
101	Funil	2	3	5
102	Funil	2	3	5
103	Funil	2	3	5
107	Funil	2	3	5
113	Funil	2	3	5
122	Funil	2	3	5
124	Funil	2	3	5
131	Funil	2	3	5
9	Camargos	2	3	6
16	Camargos	2	3	6
19	Camargos	2	3	6
30	Itutinga_1	2	3	6
32	Itutinga_1	2	3	6
48	Itutinga_1	2	3	6
51	Itutinga_1	2	3	6
52	Itutinga_1	2	3	6
53	Itutinga_1	2	3	6
56	Itutinga_1	2	3	6
57	Itutinga_1	2	3	6
66	Itutinga_2	2	3	6
77	Itutinga_2	2	3	6
104	Funil	2	3	6
106	Funil	2	3	6
108	Funil	2	3	6
123	Funil	2	3	6
136	Funil	2	3	6
138	Funil	2	3	6
5	Camargos	2	4	7
6	Camargos	2	4	7
7	Camargos	2	4	7
10	Camargos	2	4	7
12	Camargos	2	4	7
22	Itutinga_1	2	4	7
23	Itutinga_1	2	4	7
31	Itutinga_1	2	4	7
54	Itutinga_1	2	4	7
71	Itutinga_2	2	4	7
82	Itutinga_2	2	4	7
135	Funil	2	4	7

MLE Hierarchical Cluster Membership

11	Camargos	2	4	8
21	Itutinga_1	2	4	8
27	Itutinga_1	2	4	8
29	Itutinga_1	2	4	8
35	Itutinga_1	2	4	8
37	Itutinga_1	2	4	8
46	Itutinga_1	2	4	8
47	Itutinga_1	2	4	8
76	Itutinga_2	2	4	8
86	Itutinga_2	2	4	8
89	Itutinga_2	2	4	8
90	Itutinga_2	2	4	8
112	Funil	2	4	8
117	Funil	2	4	8
121	Funil	2	4	8
125	Funil	2	4	8
126	Funil	2	4	8
127	Funil	2	4	8
129	Funil	2	4	8
