# Supplementary material for: A fractal pattern of hierarchical genetic population structure in mixed stocks across fish segregated by dams revealed by genomic resources for curimba Prochilodus lineatus
Source: J Fish Biol. 2025 Nov 23;108(4):1029–46. doi: 10.1111/jfb.70278 (PMC13193354; doi:10.1111/jfb.70278)
Supplement: Supplementary file 1 — Microsatellite DNA sequences and primers presented in the plain text format (txt) for Prochilodus lineatus. The order of columns is ‘Locus’; ‘Original fasta label’; ‘Motif’; ‘Repeats’; ‘5′‐flank’; ‘3′‐flank’; ‘F‐primer’; ‘F‐Tm’; ‘R‐Primer’; ‘R‐Tm’; ‘PCR product’; ‘Product length’; ‘Location’; ‘Scaffold or contig label in the newly presented assembly, when mapped’. This file is available at fisgshare under doi: 10.6084/m9.figshare.26848654. https://doi.org/10.6084/m9.figshare.26848654. Supplementary File 2. List of DNA sequences presented in the fasta format for contigs from the original assembly from where microsatellite loci for Prochilodus lineatus were originally characterized. This file is available at fisgshare under doi: 10.6084/m9.figshare.26848015 https://doi.org/10.6084/m9.figshare.26848015. Supplementary File 3. Prochilodus lineatus population samples, fish IDs and their rearrangements based on the first‐, second‐ and third‐order maximum likelihood estimation (MLE)‐defined clusters. This file is available at fisgshare under doi: 10.6084/m9.figshare.26871031. https://doi.org/10.6084/m9.figshare.26871031. Supplementary File 4. Supplementary tables, including log‐likelihood values of the clustering analysis for panmixia and genetic diversity estimates for Prochilodus lineatus maximum likelihood estimation (MLE)‐defined clusters from the first, second and third orders of hierarchical analysis. This file is available at fisgshare under doi: 10.6084/m9.figshare.27020857. https://doi.org/10.6084/m9.figshare.27020857. Supplementary File 5. Hardy–Weinberg exact tests for maximum likelihood estimation (MLE)‐defined clusters of Prochilodus lineatus from the first, second and third orders of hierarchical analysis. This file is available at fisgshare under doi: 10.6084/m9.figshare.27020869. https://doi.org/10.6084/m9.figshare.27020869. Supplementary Figures 1–5. Supplementary Figures 1–5 are presented in a single portable document format (pdf) fil [file JFB-108-1029-s001.zip › Supplementary File 4/Supplementary_Tables_CCor.pdf]

Supplementary Tables

Supplementary Table 1: Direct comparison of log likelihood values of clustering analysis, for chosen K, according to D_{LK2}, against K=1 (panmixia) in *Prochilodus lineatus* from the Upper Grande River, MG. Note the very high rate of convergence of estimates across replicate runs (N=100) .

**Hierarchical order**	**Subject cluster**	**K**	**Log likelihood average**	**Log likelihood minimum**	**Log likelihood maximum**
1st	Total sample	1	-690.63	-690.63	-690.63
		2	-655.47	-655.80	-655.40
2nd	1	1	-381.23	-381.23	-381.23
		2	-354.19	-354.64	-354.19
	2	1	-374.69	-374.69	-374.69
		2	-348.27	-348.27	-348.27
3rd	1	1	-227.08	-227.08	-227.08
		2	-207.30	-207.35	-207.30
	2	1	-233.94	-233.94	-233.94
		2	-214.17	-214.17	-214.17
	3	1	-217.25	-217.25	-217.25
		2	-196.96	-196.96	-196.96
	4	1	-194.87	-194.87	-194.87
		2	-176.95	-176.95	-176.95

Analyses of clusters defined by hierarchical population genetic structure analysis

Supplementary Table 2: Average intrapopulation diversity estimates among clusters.

Level	N_A	H_O	H_E	F
**1st order (2 clusters)**	19	0.450	0.893	0.500
**2nd order (4 clusters)**	14	0.450	0.869	0.486
**3rd order (8 clusters)**	10	0.451	0.839	0.473

N_A=Number of alleles; H_O=Observed heterozygosity; H_E=Expected heterozygosity; F=inbreeding coefficient

Supplementary Table 3: Pairwise fixation index G''_{ST} (below diagonal) and Jost's genetic distance D (above diagonal) for clusters defined from the 1st order analysis. Overall G''_{ST}=0.255; Overall D=0.236.

	Cluster 1	Cluster 2
**Cluster 1**	-	0.237**
**Cluster 2**	0.255**	-

**p<0.001

Supplementary Table 4: Pairwise fixation index G''_{ST} (below diagonal) and Jost's genetic distance D (above diagonal) for clusters defined from the 2st order analysis. Overall G''_{ST}=0.318; Overall D=0.294.

	**Cluster 1**	**Cluster 2**	**Cluster 3**	**Cluster 4**
**Cluster 1**	-	0.224**	0.319**	0.337**
**Cluster 2**	0.244**	-	0.314**	0.338**
**Cluster 3**	0.344**	0.339**	-	0.236**
**Cluster 4**	0.363**	0.366**	0.258**	-

**p<0.001

Supplementary Table 5: Pairwise fixation index G''_{ST} (below diagonal) and Jost's genetic distance D (above diagonal) for clusters defined from the 3st order analysis. Overall G''_{ST}=0.324; Overall D=0.298.

	**Cluster 1**	**Cluster 2**	**Cluster 3**	**Cluster 4**	**Cluster 5**	**Cluster 6**	**Cluster 7**	**Cluster 8**
**Cluster 1**	-	0.518**	0.271**	0.259**	0.333**	0.306**	0.469**	0.463**
**Cluster 2**	0.550**	-	0.372**	0.217**	0.335**	0.281**	0.247**	0.257*
**Cluster 3**	0.295**	0.400**	-	0.231**	0.262**	0.295**	0.286**	0.357**
**Cluster 4**	0.282**	0.236*	0.251**	-	0.160*	0.135*	0.328**	0.162*
**Cluster 5**	0.363**	0.364**	0.286**	0.176*	-	0.132*	0.346**	0.396**
**Cluster 6**	0.331**	0.304*	0.318**	0.148*	0.145*	-	0.161*	0.333**
**Cluster 7**	0.501**	0.271**	0.312**	0.354**	0.376**	0.178*	-	0.425**
**Cluster 8**	0.497**	0.283*	0.386**	0.179**	0.429**	0.361**	0.458**	-

*p<0.05; **p<0.001
