# Supplementary material for: A fractal pattern of hierarchical genetic population structure in mixed stocks across fish segregated by dams revealed by genomic resources for curimba Prochilodus lineatus
Source: J Fish Biol. 2025 Nov 23;108(4):1029–46. doi: 10.1111/jfb.70278 (PMC13193354; doi:10.1111/jfb.70278)
Supplement: Supplementary file 1 — Microsatellite DNA sequences and primers presented in the plain text format (txt) for Prochilodus lineatus. The order of columns is ‘Locus’; ‘Original fasta label’; ‘Motif’; ‘Repeats’; ‘5′‐flank’; ‘3′‐flank’; ‘F‐primer’; ‘F‐Tm’; ‘R‐Primer’; ‘R‐Tm’; ‘PCR product’; ‘Product length’; ‘Location’; ‘Scaffold or contig label in the newly presented assembly, when mapped’. This file is available at fisgshare under doi: 10.6084/m9.figshare.26848654. https://doi.org/10.6084/m9.figshare.26848654. Supplementary File 2. List of DNA sequences presented in the fasta format for contigs from the original assembly from where microsatellite loci for Prochilodus lineatus were originally characterized. This file is available at fisgshare under doi: 10.6084/m9.figshare.26848015 https://doi.org/10.6084/m9.figshare.26848015. Supplementary File 3. Prochilodus lineatus population samples, fish IDs and their rearrangements based on the first‐, second‐ and third‐order maximum likelihood estimation (MLE)‐defined clusters. This file is available at fisgshare under doi: 10.6084/m9.figshare.26871031. https://doi.org/10.6084/m9.figshare.26871031. Supplementary File 4. Supplementary tables, including log‐likelihood values of the clustering analysis for panmixia and genetic diversity estimates for Prochilodus lineatus maximum likelihood estimation (MLE)‐defined clusters from the first, second and third orders of hierarchical analysis. This file is available at fisgshare under doi: 10.6084/m9.figshare.27020857. https://doi.org/10.6084/m9.figshare.27020857. Supplementary File 5. Hardy–Weinberg exact tests for maximum likelihood estimation (MLE)‐defined clusters of Prochilodus lineatus from the first, second and third orders of hierarchical analysis. This file is available at fisgshare under doi: 10.6084/m9.figshare.27020869. https://doi.org/10.6084/m9.figshare.27020869. Supplementary Figures 1–5. Supplementary Figures 1–5 are presented in a single portable document format (pdf) fil [file JFB-108-1029-s001.zip › Supplementary File 5/EHW-ALL-ORDERS_CC.pdf]

Results from GENEPOP 1st ORDER

Hardy-Weinberg test

Number of populations detected: 2
Number of loci detected: 15

Estimation of exact P-Values by the Markov chain method.

Markov chain parameters for all tests:
Dememorization: 1000
Batches: 100
Iterations per batch: 1000
Hardy Weinberg: Probability test
****

===
Results by locus
===

Locus "Prol01"

Fis estimates

POP	P-val	S.E.	W&C	R&H	Steps
Cluster1	0.0000	0.0000	0.6458	0.4288	3757 switches
Cluster2	0.0000	0.0000	0.7332	0.6322	2095 switches

All (Fisher's method):
Chi2: > 63.7695
Df : 4
Prob : < 4.67e-13

Locus "Prol02"

Fis estimates

POP	P-val	S.E.	W&C	R&H	Steps
Cluster1	0.0000	0.0000	0.3957	0.2508	4172 switches
Cluster2	0.0000	0.0000	0.4312	0.3051	2649 switches

All (Fisher's method):
Chi2: > 63.7695
Df : 4
Prob : < 4.67e-13

Locus "Prol03"

Fis estimates

```
                              ---------------
POP          P-val   S.E.    W&C     R&H     Steps
----------- ------- ------- ------- ------- ------
Cluster1    0.0000  0.0000  0.5271  0.4438  2571 switches
Cluster2    0.0000  0.0000  0.5248  0.4174  3261 switches

All (Fisher's method):
 Chi2:     > 63.7695
 Df   :    4
 Prob :     < 4.67e-13

Locus "Prol04"
----------------------------------------
                               Fis estimates
                              ---------------
POP          P-val   S.E.    W&C     R&H     Steps
----------- ------- ------- ------- ------- ------
Cluster1    0.0000  0.0000  0.6822  0.6021  2398 switches
Cluster2    0.0000  0.0000  0.5121  0.3710  3897 switches

All (Fisher's method):
 Chi2:     > 63.7695
 Df   :    4
 Prob :     < 4.67e-13

Locus "Prol05"
----------------------------------------
                               Fis estimates
                              ---------------
POP          P-val   S.E.    W&C     R&H     Steps
----------- ------- ------- ------- ------- ------
Cluster1    0.0000  0.0000  0.5113  0.3926  1893 switches
Cluster2    0.0000  0.0000  0.4599  0.3475  1475 switches

All (Fisher's method):
 Chi2:     > 63.7695
 Df   :    4
 Prob :     < 4.67e-13

Locus "Prol06"
----------------------------------------
                               Fis estimates
                              ---------------
POP          P-val   S.E.    W&C     R&H     Steps
----------- ------- ------- ------- ------- ------
Cluster1    0.0000  0.0000  0.8389  0.6725  7042 switches
Cluster2    0.0000  0.0000  0.6251  0.4763  4156 switches

All (Fisher's method):
 Chi2:     > 63.7695
 Df   :    4
 Prob :     < 4.67e-13
```

```
Locus "Prol07"
----------------------------------------
                     Fis estimates
                    ---------------
POP        P-val   S.E.    W&C     R&H     Steps
----------- ------- ------- ------- ------- ------
Cluster1    0.0420  0.0155   0.0719  0.0969   3547 switches
Cluster2    0.1238  0.0209   0.0353  0.0652   3672 switches

All (Fisher's method):
 Chi2:    10.5188
 Df   :    4
 Prob :    0.032539

Locus "Prol08"
----------------------------------------
                     Fis estimates
                    ---------------
POP        P-val   S.E.    W&C     R&H     Steps
----------- ------- ------- ------- ------- ------
Cluster1    0.0000  0.0000   0.4413  0.2695   5528 switches
Cluster2    0.0000  0.0000   0.3604  0.2105   6273 switches

All (Fisher's method):
 Chi2:     > 63.7695
 Df   :    4
 Prob :     < 4.67e-13

Locus "Prol10"
----------------------------------------
                     Fis estimates
                    ---------------
POP        P-val   S.E.    W&C     R&H     Steps
----------- ------- ------- ------- ------- ------
Cluster1    0.0000  0.0000   0.1199  0.1124   2084 switches
Cluster2    0.0012  0.0012   0.0564  0.0497   2485 switches

All (Fisher's method):
 Chi2:     > 45.2701
 Df   :    4
 Prob :     < 3.49e-09

Locus "Prol11"
----------------------------------------
                     Fis estimates
                    ---------------
POP        P-val   S.E.    W&C     R&H     Steps
----------- ------- ------- ------- ------- ------
Cluster1    0.0000  0.0000   0.6468  0.5842   3236 switches
Cluster2    0.0000  0.0000   0.4835  0.3144   3358 switches
```

All (Fisher's method):
Chi2: > 63.7695
Df : 4
Prob : < 4.67e-13

Locus "Prol12"

POP	P-val	S.E.	Fis estimates W&C	R&H	Steps
Cluster1	0.0000	0.0000	0.7654	0.6781	4187 switches
Cluster2	0.0000	0.0000	0.7296	0.6563	3913 switches

All (Fisher's method):
Chi2: > 63.7695
Df : 4
Prob : < 4.67e-13

Locus "Prol19"

POP	P-val	S.E.	Fis estimates W&C	R&H	Steps
Cluster1	0.0000	0.0000	0.7902	0.6540	6772 switches
Cluster2	0.0000	0.0000	0.7582	0.6639	5421 switches

All (Fisher's method):
Chi2: > 63.7695
Df : 4
Prob : < 4.67e-13

Locus "Prol53"

POP	P-val	S.E.	Fis estimates W&C	R&H	Steps
Cluster1	0.0000	0.0000	0.6995	0.2772	4251 switches
Cluster2	0.0000	0.0000	0.5957	0.2007	3574 switches

All (Fisher's method):
Chi2: > 63.7695
Df : 4
Prob : < 4.67e-13

Locus "Prol54"

POP	P-val	S.E.	Fis estimates W&C	R&H	Steps

```
----------- ------- ------- ------- ------- ------
Cluster1    0.0000  0.0000   0.5911  0.3778   1812 switches
Cluster2    0.0000  0.0000   0.4797  0.4107   2463 switches

All (Fisher's method):
 Chi2:     > 63.7695
 Df   :    4
 Prob :     < 4.67e-13

Locus "Prol57"
-----------------------------------------
                         Fis estimates
                        ---------------
POP         P-val   S.E.    W&C     R&H     Steps
----------- ------- ------- ------- ------- ------
Cluster1    0.0000  0.0000   0.3171  0.2040   2366 switches
Cluster2    0.0000  0.0000   0.3445  0.2217   2430 switches

All (Fisher's method):
 Chi2:     > 63.7695
 Df   :    4
 Prob :     < 4.67e-13

==========================================
    Results by population
==========================================


Pop : Cluster1
-----------------------------------------
                         Fis estimates
                        ---------------
locus       P-val   S.E.    W&C     R&H     Steps
----------- ------- ------- ------- ------- ------
Prol01      0.0000  0.0000   0.6458  0.4288   3757 switches
Prol02      0.0000  0.0000   0.3957  0.2508   4172 switches
Prol03      0.0000  0.0000   0.5271  0.4438   2571 switches
Prol04      0.0000  0.0000   0.6822  0.6021   2398 switches
Prol05      0.0000  0.0000   0.5113  0.3926   1893 switches
Prol06      0.0000  0.0000   0.8389  0.6725   7042 switches
Prol07      0.0420  0.0155   0.0719  0.0969   3547 switches
Prol08      0.0000  0.0000   0.4413  0.2695   5528 switches
Prol10      0.0000  0.0000   0.1199  0.1124   2084 switches
Prol11      0.0000  0.0000   0.6468  0.5842   3236 switches
Prol12      0.0000  0.0000   0.7654  0.6781   4187 switches
Prol19      0.0000  0.0000   0.7902  0.6540   6772 switches
Prol53      0.0000  0.0000   0.6995  0.2772   4251 switches
Prol54      0.0000  0.0000   0.5911  0.3778   1812 switches
Prol57      0.0000  0.0000   0.3171  0.2040   2366 switches

All (Fisher's method):
 Chi2 :     > 328.7021
```

Df : 30
Prob : < 0.00e+00

Pop : Cluster2

Fis estimates

----

locus	P-val	S.E.	W&C	R&H	Steps
Prol01	0.0000	0.0000	0.7332	0.6322	2095 switches
Prol02	0.0000	0.0000	0.4312	0.3051	2649 switches
Prol03	0.0000	0.0000	0.5248	0.4174	3261 switches
Prol04	0.0000	0.0000	0.5121	0.3710	3897 switches
Prol05	0.0000	0.0000	0.4599	0.3475	1475 switches
Prol06	0.0000	0.0000	0.6251	0.4763	4156 switches
Prol07	0.1238	0.0209	0.0353	0.0652	3672 switches
Prol08	0.0000	0.0000	0.3604	0.2105	6273 switches
Prol10	0.0012	0.0012	0.0564	0.0497	2485 switches
Prol11	0.0000	0.0000	0.4835	0.3144	3358 switches
Prol12	0.0000	0.0000	0.7296	0.6563	3913 switches
Prol19	0.0000	0.0000	0.7582	0.6639	5421 switches
Prol53	0.0000	0.0000	0.5957	0.2007	3574 switches
Prol54	0.0000	0.0000	0.4797	0.4107	2463 switches
Prol57	0.0000	0.0000	0.3445	0.2217	2430 switches

All (Fisher's method):
Chi2 : > 316.9000
Df : 30
Prob : < 0.00e+00

==

All locus, all populations

==

All (Fisher's method) :
Chi2 : > 645.6021
Df : 60
Prob : < 0.00e+00

==

Results from GENEPOP 2nd ORDER

Hardy-Weinberg test

Number of populations detected: 4
Number of loci detected: 15

Estimation of exact P-Values by the Markov chain method.

Markov chain parameters for all tests:
Dememorization: 1000
Batches: 100
Iterations per batch: 1000
Hardy Weinberg: Probability test
****

==
Results by locus
==

Locus "Prol01"

POP	P-val	S.E.	Fis estimates W&C	Fis estimates R&H	Steps
Cluster1	0.0000	0.0000	0.5540	0.4241	3151 switches
Cluster2	0.0000	0.0000	0.7469	0.5064	4978 switches
Cluster3	0.0000	0.0000	0.7604	0.6531	2005 switches
Cluster4	0.0000	0.0000	0.6696	0.4640	4086 switches

All (Fisher's method):
Chi2: > 127.5391
Df : 8
Prob : < 9.15e-24

Locus "Prol02"

POP	P-val	S.E.	Fis estimates W&C	Fis estimates R&H	Steps
Cluster1	0.0000	0.0000	0.4105	0.2517	4597 switches
Cluster2	0.0000	0.0000	0.3795	0.2153	2470 switches
Cluster3	0.0000	0.0000	0.4351	0.2786	1396 switches
Cluster4	0.0000	0.0000	0.4046	0.2523	1523 switches

All (Fisher's method):
Chi2: > 127.5391
Df : 8
Prob : < 9.15e-24

Locus "Prol03"

Fis estimates

POP	P-val	S.E.	W&C	R&H	Steps
Cluster1	0.0000	0.0000	0.5190	0.4448	2398 switches
Cluster2	0.0000	0.0000	0.5302	0.4698	3313 switches
Cluster3	0.0000	0.0000	0.5579	0.4181	2776 switches
Cluster4	0.0000	0.0000	0.4562	0.3526	2889 switches

All (Fisher's method):
Chi2: > 127.5391
Df : 8
Prob : < 9.15e-24

Locus "Prol04"

Fis estimates

POP	P-val	S.E.	W&C	R&H	Steps
Cluster1	0.0000	0.0000	0.7614	0.7098	3383 switches
Cluster2	0.0000	0.0000	0.5970	0.4704	1996 switches
Cluster3	0.0000	0.0000	0.4455	0.3782	3105 switches
Cluster4	0.0000	0.0000	0.5912	0.4650	4047 switches

All (Fisher's method):
Chi2: > 127.5391
Df : 8
Prob : < 9.15e-24

Locus "Prol05"

Fis estimates

POP	P-val	S.E.	W&C	R&H	Steps
Cluster1	0.0000	0.0000	0.4358	0.2781	1229 switches
Cluster2	0.0000	0.0000	0.5798	0.3794	2514 switches
Cluster3	0.0000	0.0000	0.4351	0.3558	2975 switches
Cluster4	0.0000	0.0000	0.4706	0.3259	1228 switches

All (Fisher's method):
Chi2: > 127.5391
Df : 8
Prob : < 9.15e-24

Locus "Prol06"

Fis estimates

```
POP         P-val   S.E.    W&C     R&H     Steps
----------- ------- ------- ------- ------- ------
Cluster1    0.0000  0.0000   0.8006  0.6745   4633 switches
Cluster2    0.0000  0.0000   0.8729  0.7771  12244 switches
Cluster3    0.0000  0.0000   0.5600  0.4220   5171 switches
Cluster4    0.0000  0.0000   0.6849  0.5952  10575 switches

All (Fisher's method):
 Chi2:     > 127.5391
 Df   :    8
 Prob :     < 9.15e-24

Locus "Prol07"
----------------------------------------
                          Fis estimates
                         ---------------
POP         P-val   S.E.    W&C     R&H     Steps
----------- ------- ------- ------- ------- ------
Cluster1    0.1728  0.0258  -0.0065 -0.0035   2661 switches
Cluster2    0.1919  0.0191   0.1064  0.2444   6316 switches
Cluster3    0.1087  0.0170  -0.0056 -0.0265   3504 switches
Cluster4    0.7234  0.0224   0.0612  0.0823   3548 switches

All (Fisher's method):
 Chi2:    11.8991
 Df   :    8
 Prob :    0.155764

Locus "Prol08"
----------------------------------------
                          Fis estimates
                         ---------------
POP         P-val   S.E.    W&C     R&H     Steps
----------- ------- ------- ------- ------- ------
Cluster1    0.0000  0.0000   0.3883  0.2011   4095 switches
Cluster2    0.0000  0.0000   0.4947  0.3379   6039 switches
Cluster3    0.0000  0.0000   0.4001  0.2391   3862 switches
Cluster4    0.0118  0.0052   0.2778  0.1556   6534 switches

All (Fisher's method):
 Chi2:     > 104.5387
 Df   :    8
 Prob :     < 5.03e-19

Locus "Prol10"
----------------------------------------
                          Fis estimates
                         ---------------
POP         P-val   S.E.    W&C     R&H     Steps
----------- ------- ------- ------- ------- ------
Cluster1    0.0000  0.0000   0.0290  0.0152   1371 switches
Cluster2    0.0019  0.0019   0.2177  0.1506   1744 switches
```

```
Cluster3    0.0225  0.0102   0.0892  0.0487   2033 switches
Cluster4    0.0376  0.0162  -0.0127 -0.0035    964 switches (low!)

All (Fisher's method):
 Chi2:     > 58.5304
 Df   :    8
 Prob :     < 9.05e-10

Locus "Prol11"
----------------------------------------
                     Fis estimates
                     ---------------
POP         P-val   S.E.    W&C     R&H     Steps
----------- ------- ------- ------- ------- ------
Cluster1    0.0000  0.0000   0.7500  0.7409   3665 switches
Cluster2    0.0000  0.0000   0.5098  0.3517   2522 switches
Cluster3    0.0000  0.0000   0.3756  0.2255   2232 switches
Cluster4    0.0000  0.0000   0.6052  0.3523   3189 switches

All (Fisher's method):
 Chi2:     > 127.5391
 Df   :    8
 Prob :     < 9.15e-24

Locus "Prol12"
----------------------------------------
                     Fis estimates
                     ---------------
POP         P-val   S.E.    W&C     R&H     Steps
----------- ------- ------- ------- ------- ------
Cluster1    0.0000  0.0000   0.7496  0.6529   3284 switches
Cluster2    0.0000  0.0000   0.7736  0.7309   3690 switches
Cluster3    0.0000  0.0000   0.6368  0.5854   3437 switches
Cluster4    0.0000  0.0000   0.8329  0.7716   3239 switches

All (Fisher's method):
 Chi2:     > 127.5391
 Df   :    8
 Prob :     < 9.15e-24

Locus "Prol19"
----------------------------------------
                     Fis estimates
                     ---------------
POP         P-val   S.E.    W&C     R&H     Steps
----------- ------- ------- ------- ------- ------
Cluster1    0.0000  0.0000   0.7226  0.6134   5776 switches
Cluster2    0.0000  0.0000   0.8466  0.7894  10397 switches
Cluster3    0.0000  0.0000   0.8272  0.7219   6155 switches
Cluster4    0.0000  0.0000   0.6255  0.4528   5240 switches

All (Fisher's method):
```

Chi2: > 127.5391
Df : 8
Prob : < 9.15e-24

Locus "Prol53"

Fis estimates

POP	P-val	S.E.	W&C	R&H	Steps
Cluster1	0.0000	0.0000	0.6688	0.2681	2101 switches
Cluster2	0.0000	0.0000	0.7232	0.4872	7883 switches
Cluster3	0.0000	0.0000	0.4926	0.1311	2517 switches
Cluster4	0.0000	0.0000	0.7264	0.2484	3768 switches

All (Fisher's method):
Chi2: > 127.5391
Df : 8
Prob : < 9.15e-24

Locus "Prol54"

Fis estimates

POP	P-val	S.E.	W&C	R&H	Steps
Cluster1	0.0000	0.0000	0.6859	0.4614	1747 switches
Cluster2	0.0000	0.0000	0.4542	0.2684	2450 switches
Cluster3	0.0000	0.0000	0.4934	0.4211	2091 switches
Cluster4	0.0000	0.0000	0.4460	0.3702	2511 switches

All (Fisher's method):
Chi2: > 127.5391
Df : 8
Prob : < 9.15e-24

Locus "Prol57"

Fis estimates

POP	P-val	S.E.	W&C	R&H	Steps
Cluster1	0.0000	0.0000	0.2863	0.2144	2459 switches
Cluster2	0.0000	0.0000	0.3381	0.1753	2147 switches
Cluster3	0.0000	0.0000	0.3023	0.1904	2611 switches
Cluster4	0.0000	0.0000	0.3953	0.2518	1458 switches

All (Fisher's method):
Chi2: > 127.5391
Df : 8
Prob : < 9.15e-24

==
Results by population
==

Pop : Cluster1

Fis estimates

locus	P-val	S.E.	W&C	R&H	Steps
Prol01	0.0000	0.0000	0.5540	0.4241	3151 switches
Prol02	0.0000	0.0000	0.4105	0.2517	4597 switches
Prol03	0.0000	0.0000	0.5190	0.4448	2398 switches
Prol04	0.0000	0.0000	0.7614	0.7098	3383 switches
Prol05	0.0000	0.0000	0.4358	0.2781	1229 switches
Prol06	0.0000	0.0000	0.8006	0.6745	4633 switches
Prol07	0.1728	0.0258	-0.0065	-0.0035	2661 switches
Prol08	0.0000	0.0000	0.3883	0.2011	4095 switches
Prol10	0.0000	0.0000	0.0290	0.0152	1371 switches
Prol11	0.0000	0.0000	0.7500	0.7409	3665 switches
Prol12	0.0000	0.0000	0.7496	0.6529	3284 switches
Prol19	0.0000	0.0000	0.7226	0.6134	5776 switches
Prol53	0.0000	0.0000	0.6688	0.2681	2101 switches
Prol54	0.0000	0.0000	0.6859	0.4614	1747 switches
Prol57	0.0000	0.0000	0.2863	0.2144	2459 switches

All (Fisher's method):
Chi2 : > 325.8728
Df : 30
Prob : < 0.00e+00

Pop : Cluster2

Fis estimates

locus	P-val	S.E.	W&C	R&H	Steps
Prol01	0.0000	0.0000	0.7469	0.5064	4978 switches
Prol02	0.0000	0.0000	0.3795	0.2153	2470 switches
Prol03	0.0000	0.0000	0.5302	0.4698	3313 switches
Prol04	0.0000	0.0000	0.5970	0.4704	1996 switches
Prol05	0.0000	0.0000	0.5798	0.3794	2514 switches
Prol06	0.0000	0.0000	0.8729	0.7771	12244 switches
Prol07	0.1919	0.0191	0.1064	0.2444	6316 switches
Prol08	0.0000	0.0000	0.4947	0.3379	6039 switches
Prol10	0.0019	0.0019	0.2177	0.1506	1744 switches
Prol11	0.0000	0.0000	0.5098	0.3517	2522 switches
Prol12	0.0000	0.0000	0.7736	0.7309	3690 switches
Prol19	0.0000	0.0000	0.8466	0.7894	10397 switches
Prol53	0.0000	0.0000	0.7232	0.4872	7883 switches
Prol54	0.0000	0.0000	0.4542	0.2684	2450 switches

Prol57	0.0000	0.0000	0.3381	0.1753	2147 switches

All (Fisher's method):
Chi2 : > 315.1280
Df : 30
Prob : < 0.00e+00

Pop : Cluster3

Fis estimates

locus	P-val	S.E.	W&C	R&H	Steps
Prol01	0.0000	0.0000	0.7604	0.6531	2005 switches
Prol02	0.0000	0.0000	0.4351	0.2786	1396 switches
Prol03	0.0000	0.0000	0.5579	0.4181	2776 switches
Prol04	0.0000	0.0000	0.4455	0.3782	3105 switches
Prol05	0.0000	0.0000	0.4351	0.3558	2975 switches
Prol06	0.0000	0.0000	0.5600	0.4220	5171 switches
Prol07	0.1087	0.0170	-0.0056	-0.0265	3504 switches
Prol08	0.0000	0.0000	0.4001	0.2391	3862 switches
Prol10	0.0225	0.0102	0.0892	0.0487	2033 switches
Prol11	0.0000	0.0000	0.3756	0.2255	2232 switches
Prol12	0.0000	0.0000	0.6368	0.5854	3437 switches
Prol19	0.0000	0.0000	0.8272	0.7219	6155 switches
Prol53	0.0000	0.0000	0.4926	0.1311	2517 switches
Prol54	0.0000	0.0000	0.4934	0.4211	2091 switches
Prol57	0.0000	0.0000	0.3023	0.1904	2611 switches

All (Fisher's method):
Chi2 : > 311.3679
Df : 30
Prob : < 0.00e+00

Pop : Cluster4

Fis estimates

locus	P-val	S.E.	W&C	R&H	Steps
Prol01	0.0000	0.0000	0.6696	0.4640	4086 switches
Prol02	0.0000	0.0000	0.4046	0.2523	1523 switches
Prol03	0.0000	0.0000	0.4562	0.3526	2889 switches
Prol04	0.0000	0.0000	0.5912	0.4650	4047 switches
Prol05	0.0000	0.0000	0.4706	0.3259	1228 switches
Prol06	0.0000	0.0000	0.6849	0.5952	10575 switches
Prol07	0.7234	0.0224	0.0612	0.0823	3548 switches
Prol08	0.0118	0.0052	0.2778	0.1556	6534 switches
Prol10	0.0376	0.0162	-0.0127	-0.0035	964 switches (low!)
Prol11	0.0000	0.0000	0.6052	0.3523	3189 switches
Prol12	0.0000	0.0000	0.8329	0.7716	3239 switches
Prol19	0.0000	0.0000	0.6255	0.4528	5240 switches

```
Prol53      0.0000  0.0000   0.7264  0.2484   3768 switches
Prol54      0.0000  0.0000   0.4460  0.3702   2511 switches
Prol57      0.0000  0.0000   0.3953  0.2518   1458 switches

All (Fisher's method):
 Chi2 :     > 292.4047
 Df   :    30
 Prob :     < 8.41e-45
=========================================
 All locus, all populations
=========================================
All (Fisher's method) :
 Chi2 :     > 1244.7733
 Df   :    120
 Prob :    High. sign.

================================================
```

Results from GENEPOP 3rd ORDER

Hardy-Weinberg test

Number of populations detected: 8
Number of loci detected: 15

Estimation of exact P-Values by the Markov chain method.

Markov chain parameters for all tests:
Dememorization: 1000
Batches: 100
Iterations per batch: 1000
Hardy Weinberg: Probability test
****

==
Results by locus
==

Locus "Prol01"

POP	P-val	S.E.	Fis estimates W&C	Fis estimates R&H	Steps
Cluster1	0.0000	0.0000	0.4090	0.2419	2918 switches
Cluster2	0.0000	0.0000	0.6560	0.4691	3210 switches
Cluster3	0.0000	0.0000	0.8140	0.5377	6288 switches
Cluster4	0.0000	0.0000	0.6581	0.3423	4946 switches
Cluster5	0.0000	0.0000	0.6959	0.5734	2284 switches
Cluster6	0.0000	0.0000	0.8033	0.6179	3179 switches
Cluster7	0.0288	0.0044	0.4435	0.5229	7169 switches
Cluster8	0.0000	0.0000	0.7581	0.6017	3364 switches

All (Fisher's method):
Chi2: > 230.2867
Df : 16
Prob : < 5.59e-40

Locus "Prol02"

POP	P-val	S.E.	Fis estimates W&C	Fis estimates R&H	Steps
Cluster1	0.0222	0.0053	0.2258	0.1265	3310 switches
Cluster2	0.0000	0.0000	0.5745	0.4768	5735 switches
Cluster3	0.0609	0.0131	0.2628	0.2058	2220 switches
Cluster4	0.0000	0.0000	0.4633	0.2660	5932 switches
Cluster5	0.0000	0.0000	0.5415	0.3753	1483 switches

```
Cluster6    0.0609  0.0123   0.2982  0.1329   3304 switches
Cluster7    0.0025  0.0014   0.4410  0.2667   4679 switches
Cluster8    0.0000  0.0000   0.3822  0.3013   1627 switches

All (Fisher's method):
 Chi2:     > 158.3591
 Df   :    16
 Prob :     < 1.74e-25

Locus "Prol03"
----------------------------------------
                      Fis estimates
                     ---------------
POP         P-val   S.E.    W&C     R&H     Steps
----------- ------- ------- ------- ------- ------
Cluster1    0.0000  0.0000   0.6526  0.6320   3040 switches
Cluster2    0.0000  0.0000   0.4010  0.1913   1180 switches
Cluster3    0.0000  0.0000   0.7456  0.6406   4838 switches
Cluster4    0.0113  0.0063   0.3319  0.2357   1825 switches
Cluster5    0.0000  0.0000   0.5704  0.4572   2830 switches
Cluster6    0.0000  0.0000   0.5270  0.3527   2723 switches
Cluster7    0.0295  0.0079   0.2125  0.0995   3254 switches
Cluster8    0.0000  0.0000   0.6061  0.5736   3816 switches

All (Fisher's method):
 Chi2:     > 207.3211
 Df   :    16
 Prob :     < 2.62e-35

Locus "Prol04"
----------------------------------------
                      Fis estimates
                     ---------------
POP         P-val   S.E.    W&C     R&H     Steps
----------- ------- ------- ------- ------- ------
Cluster1    0.0000  0.0000   0.7685  0.6269   2978 switches
Cluster2    0.0000  0.0000   0.7495  0.6687   3061 switches
Cluster3    0.0000  0.0000   0.5136  0.3440   1297 switches
Cluster4    0.0000  0.0000   0.6748  0.6248   3721 switches
Cluster5    0.0014  0.0012   0.4483  0.3113   3357 switches
Cluster6    0.0001  0.0001   0.4263  0.3819   4028 switches
Cluster7    0.0000  0.0000   0.6117  0.5531   4332 switches
Cluster8    0.0000  0.0000   0.5817  0.4349   3405 switches

All (Fisher's method):
 Chi2:     > 223.2757
 Df   :    16
 Prob :     < 1.50e-38

Locus "Prol05"
----------------------------------------
                      Fis estimates
```

POP	P-val	S.E.	W&C	R&H	Steps
Cluster1	0.0000	0.0000	0.6637	0.5913	3772 switches
Cluster2	0.0024	0.0024	0.1837	0.0887	1194 switches
Cluster3	0.0000	0.0000	0.5960	0.3781	3492 switches
Cluster4	0.0000	0.0000	0.5591	0.4724	3030 switches
Cluster5	0.0000	0.0000	0.4386	0.2613	1899 switches
Cluster6	0.0000	0.0000	0.4089	0.3183	2791 switches
Cluster7	0.0000	0.0000	0.4819	0.3000	3242 switches
Cluster8	0.0000	0.0000	0.4493	0.3292	1284 switches

All (Fisher's method):
Chi2: > 226.4242
Df : 16
Prob : < 3.43e-39

Locus "Prol06"

Fis estimates

POP	P-val	S.E.	W&C	R&H	Steps
Cluster1	0.0000	0.0000	0.7600	0.7295	4785 switches
Cluster2	0.0000	0.0000	0.8248	0.6156	5727 switches
Cluster3	0.0000	0.0000	0.8229	0.8800	15630 switches
Cluster4	0.0000	0.0000	0.9176	0.6571	11284 switches
Cluster5	0.0127	0.0034	0.3548	0.1009	5312 switches
Cluster6	0.0000	0.0000	0.7067	0.7086	12521 switches
Cluster7	0.0129	0.0025	0.5322	0.6310	8383 switches
Cluster8	0.0000	0.0000	0.8035	0.5881	20775 switches

All (Fisher's method):
Chi2: > 208.7405
Df : 16
Prob : < 1.35e-35

Locus "Prol07"

Fis estimates

POP	P-val	S.E.	W&C	R&H	Steps
Cluster1	0.0272	0.0066	0.0871	0.0455	3363 switches
Cluster2	0.8883	0.0126	-0.1055	-0.0667	2975 switches
Cluster3	0.5971	0.0182	0.1250	0.1232	4185 switches
Cluster4	0.1779	0.0125	0.0909	0.1756	5436 switches
Cluster5	0.4926	0.0239	-0.0249	-0.0355	3613 switches
Cluster6	0.0460	0.0090	0.0280	-0.0110	3646 switches
Cluster7	0.5616	0.0293	-0.0667	-0.0437	1699 switches
Cluster8	0.3523	0.0213	0.1608	0.2037	3942 switches

All (Fisher's method):
Chi2: 22.7486
Df : 16
Prob : 0.120622

Locus "Prol08"

Fis estimates

POP	P-val	S.E.	W&C	R&H	Steps
Cluster1	0.0000	0.0000	0.5504	0.4388	5776 switches
Cluster2	0.0623	0.0146	0.2147	0.1198	2657 switches
Cluster3	0.0004	0.0004	0.3735	0.2808	2834 switches
Cluster4	0.0000	0.0000	0.6308	0.5637	7184 switches
Cluster5	0.0007	0.0007	0.2867	0.2124	2804 switches
Cluster6	0.0000	0.0000	0.5103	0.2714	3447 switches
Cluster7	0.7827	0.0122	0.1064	0.0714	4561 switches
Cluster8	0.0000	0.0000	0.3712	0.2288	6982 switches

All (Fisher's method):
Chi2: > 163.7142
Df : 16
Prob : < 1.50e-26

Locus "Prol10"

Fis estimates

POP	P-val	S.E.	W&C	R&H	Steps
Cluster1	0.0113	0.0045	0.0163	-0.0021	1949 switches
Cluster2	0.0606	0.0166	0.0336	0.0417	1370 switches
Cluster3	0.0088	0.0032	0.2271	0.1550	2336 switches
Cluster4	0.0328	0.0085	0.2079	0.0815	2114 switches
Cluster5	0.0600	0.0181	0.1111	0.1370	1837 switches
Cluster6	0.1791	0.0249	0.0761	0.0410	1648 switches
Cluster7	0.1238	0.0180	0.0667	0.0317	1891 switches
Cluster8	0.1375	0.0244	-0.0685	-0.0385	1250 switches

All (Fisher's method):
Chi2: 48.0884
Df : 16
Prob : 4.60e-05

Locus "Prol11"

Fis estimates

POP	P-val	S.E.	W&C	R&H	Steps
Cluster1	0.0000	0.0000	0.7235	0.6227	4434 switches

Cluster2	0.0000	0.0000	0.7439	0.6522	4922 switches
Cluster3	0.0031	0.0011	0.5319	0.5972	9976 switches
Cluster4	0.0000	0.0000	0.4586	0.2083	2371 switches
Cluster5	0.0000	0.0000	0.4432	0.3493	4445 switches
Cluster6	0.0829	0.0180	0.2174	0.0680	3764 switches
Cluster7	0.0000	0.0000	0.5115	0.2986	1871 switches
Cluster8	0.0000	0.0000	0.6584	0.3724	3132 switches

All (Fisher's method):
Chi2: > 207.8289
Df : 16
Prob : < 2.06e-35

Locus "Prol12"

POP	P-val	S.E.	Fis estimates W&C	Fis estimates R&H	Steps
Cluster1	0.0000	0.0000	0.7073	0.5969	4041 switches
Cluster2	0.0000	0.0000	0.7766	0.6703	3719 switches
Cluster3	0.0000	0.0000	0.7802	0.8667	10387 switches
Cluster4	0.0000	0.0000	0.7429	0.6047	4322 switches
Cluster5	0.0000	0.0000	0.5520	0.4347	3877 switches
Cluster6	0.0000	0.0000	0.7037	0.6271	6474 switches
Cluster7	0.0000	0.0000	0.8710	0.8700	8166 switches
Cluster8	0.0000	0.0000	0.8061	0.8015	5116 switches

All (Fisher's method):
Chi2: > 255.0781
Df : 16
Prob : < 4.20e-45

Locus "Prol19"

POP	P-val	S.E.	Fis estimates W&C	Fis estimates R&H	Steps
Cluster1	0.0000	0.0000	0.6643	0.5586	3470 switches
Cluster2	0.0000	0.0000	0.7938	0.6567	7260 switches
Cluster3	0.0000	0.0000	0.9118	0.7857	5770 switches
Cluster4	0.0000	0.0000	0.7722	0.6147	9015 switches
Cluster5	0.0000	0.0000	0.7857	0.7185	4721 switches
Cluster6	0.0000	0.0000	0.8636	0.6993	4650 switches
Cluster7	0.2807	0.0165	0.1340	0.0833	5012 switches
Cluster8	0.0000	0.0000	0.9097	0.9938	11417 switches

All (Fisher's method):
Chi2: > 225.7343
Df : 16
Prob : < 4.74e-39

Locus "Prol53"

Fis estimates

POP	P-val	S.E.	W&C	R&H	Steps
Cluster1	0.0000	0.0000	0.8122	0.5085	7351 switches
Cluster2	0.0000	0.0000	0.5536	0.2212	3015 switches
Cluster3	0.0000	0.0000	0.7079	0.4059	7196 switches
Cluster4	0.0000	0.0000	0.7437	0.6036	6753 switches
Cluster5	0.0000	0.0000	0.8400	0.4967	9286 switches
Cluster6	0.0915	0.0204	0.2030	0.0383	2002 switches
Cluster7	0.0000	0.0000	0.6667	0.3500	5141 switches
Cluster8	0.0000	0.0000	0.7764	0.4115	7715 switches

All (Fisher's method):
Chi2: > 227.9764
Df : 16
Prob : < 1.65e-39

Locus "Prol54"

Fis estimates

POP	P-val	S.E.	W&C	R&H	Steps
Cluster1	0.0000	0.0000	0.7059	0.6181	4777 switches
Cluster2	0.0000	0.0000	0.6474	0.4473	1500 switches
Cluster3	0.0000	0.0000	0.5359	0.4462	4054 switches
Cluster4	0.0000	0.0000	0.3791	0.2241	2133 switches
Cluster5	0.0079	0.0037	0.4295	0.3365	3100 switches
Cluster6	0.0000	0.0000	0.5281	0.3579	1561 switches
Cluster7	0.0044	0.0037	0.4175	0.4194	3041 switches
Cluster8	0.0000	0.0000	0.4477	0.3386	3084 switches

All (Fisher's method):
Chi2: > 211.8266
Df : 16
Prob : < 3.19e-36

Locus "Prol57"

Fis estimates

POP	P-val	S.E.	W&C	R&H	Steps
Cluster1	0.0284	0.0094	0.1250	0.1238	1997 switches
Cluster2	0.0000	0.0000	0.4286	0.2913	2058 switches
Cluster3	0.0015	0.0015	0.3765	0.2685	2525 switches
Cluster4	0.0017	0.0012	0.3119	0.1711	3367 switches
Cluster5	0.0954	0.0150	0.2010	0.0747	2647 switches

Cluster6	0.0000	0.0000	0.3404	0.2669	3637 switches
Cluster7	0.0021	0.0011	0.5232	0.3940	5345 switches
Cluster8	0.0024	0.0024	0.3097	0.2260	1771 switches

All (Fisher's method):
Chi2: > 115.4968
Df : 16
Prob : < 4.01e-17

==
Results by population
==

Pop : Cluster1

Fis estimates
----

locus	P-val	S.E.	W&C	R&H	Steps
Prol01	0.0000	0.0000	0.4090	0.2419	2918 switches
Prol02	0.0222	0.0053	0.2258	0.1265	3310 switches
Prol03	0.0000	0.0000	0.6526	0.6320	3040 switches
Prol04	0.0000	0.0000	0.7685	0.6269	2978 switches
Prol05	0.0000	0.0000	0.6637	0.5913	3772 switches
Prol06	0.0000	0.0000	0.7600	0.7295	4785 switches
Prol07	0.0272	0.0066	0.0871	0.0455	3363 switches
Prol08	0.0000	0.0000	0.5504	0.4388	5776 switches
Prol10	0.0113	0.0045	0.0163	-0.0021	1949 switches
Prol11	0.0000	0.0000	0.7235	0.6227	4434 switches
Prol12	0.0000	0.0000	0.7073	0.5969	4041 switches
Prol19	0.0000	0.0000	0.6643	0.5586	3470 switches
Prol53	0.0000	0.0000	0.8122	0.5085	7351 switches
Prol54	0.0000	0.0000	0.7059	0.6181	4777 switches
Prol57	0.0284	0.0094	0.1250	0.1238	1997 switches

All (Fisher's method):
Chi2 : > 284.1907
Df : 30
Prob : < 3.38e-43

Pop : Cluster2

Fis estimates
----

locus	P-val	S.E.	W&C	R&H	Steps
Prol01	0.0000	0.0000	0.6560	0.4691	3210 switches
Prol02	0.0000	0.0000	0.5745	0.4768	5735 switches
Prol03	0.0000	0.0000	0.4010	0.1913	1180 switches
Prol04	0.0000	0.0000	0.7495	0.6687	3061 switches
Prol05	0.0024	0.0024	0.1837	0.0887	1194 switches

```
Prol06      0.0000  0.0000   0.8248  0.6156   5727 switches
Prol07      0.8883  0.0126  -0.1055 -0.0667   2975 switches
Prol08      0.0623  0.0146   0.2147  0.1198   2657 switches
Prol10      0.0606  0.0166   0.0336  0.0417   1370 switches
Prol11      0.0000  0.0000   0.7439  0.6522   4922 switches
Prol12      0.0000  0.0000   0.7766  0.6703   3719 switches
Prol19      0.0000  0.0000   0.7938  0.6567   7260 switches
Prol53      0.0000  0.0000   0.5536  0.2212   3015 switches
Prol54      0.0000  0.0000   0.6474  0.4473   1500 switches
Prol57      0.0000  0.0000   0.4286  0.2913   2058 switches

All (Fisher's method):
 Chi2 :     > 276.7693
 Df   :    30
 Prob :     < 9.57e-42

Pop : Cluster3
-----------------------------------------
                         Fis estimates
                        ---------------
locus       P-val   S.E.    W&C     R&H     Steps
----------- ------- ------- ------- ------- ------
Prol01      0.0000  0.0000   0.8140  0.5377   6288 switches
Prol02      0.0609  0.0131   0.2628  0.2058   2220 switches
Prol03      0.0000  0.0000   0.7456  0.6406   4838 switches
Prol04      0.0000  0.0000   0.5136  0.3440   1297 switches
Prol05      0.0000  0.0000   0.5960  0.3781   3492 switches
Prol06      0.0000  0.0000   0.8229  0.8800  15630 switches
Prol07      0.5971  0.0182   0.1250  0.1232   4185 switches
Prol08      0.0004  0.0004   0.3735  0.2808   2834 switches
Prol10      0.0088  0.0032   0.2271  0.1550   2336 switches
Prol11      0.0031  0.0011   0.5319  0.5972   9976 switches
Prol12      0.0000  0.0000   0.7802  0.8667  10387 switches
Prol19      0.0000  0.0000   0.9118  0.7857   5770 switches
Prol53      0.0000  0.0000   0.7079  0.4059   7196 switches
Prol54      0.0000  0.0000   0.5359  0.4462   4054 switches
Prol57      0.0015  0.0015   0.3765  0.2685   2525 switches

All (Fisher's method):
 Chi2 :     > 263.3518
 Df   :    30
 Prob :     < 3.93e-39

Pop : Cluster4
-----------------------------------------
                         Fis estimates
                        ---------------
locus       P-val   S.E.    W&C     R&H     Steps
----------- ------- ------- ------- ------- ------
Prol01      0.0000  0.0000   0.6581  0.3423   4946 switches
Prol02      0.0000  0.0000   0.4633  0.2660   5932 switches
Prol03      0.0113  0.0063   0.3319  0.2357   1825 switches
```

Prol04	0.0000	0.0000	0.6748	0.6248	3721 switches
Prol05	0.0000	0.0000	0.5591	0.4724	3030 switches
Prol06	0.0000	0.0000	0.9176	0.6571	11284 switches
Prol07	0.1779	0.0125	0.0909	0.1756	5436 switches
Prol08	0.0000	0.0000	0.6308	0.5637	7184 switches
Prol10	0.0328	0.0085	0.2079	0.0815	2114 switches
Prol11	0.0000	0.0000	0.4586	0.2083	2371 switches
Prol12	0.0000	0.0000	0.7429	0.6047	4322 switches
Prol19	0.0000	0.0000	0.7722	0.6147	9015 switches
Prol53	0.0000	0.0000	0.7437	0.6036	6753 switches
Prol54	0.0000	0.0000	0.3791	0.2241	2133 switches
Prol57	0.0017	0.0012	0.3119	0.1711	3367 switches

All (Fisher's method):
Chi2 : > 285.2884
Df : 30
Prob : < 2.06e-43

Pop : Cluster5

Fis estimates
----

locus	P-val	S.E.	W&C	R&H	Steps
Prol01	0.0000	0.0000	0.6959	0.5734	2284 switches
Prol02	0.0000	0.0000	0.5415	0.3753	1483 switches
Prol03	0.0000	0.0000	0.5704	0.4572	2830 switches
Prol04	0.0014	0.0012	0.4483	0.3113	3357 switches
Prol05	0.0000	0.0000	0.4386	0.2613	1899 switches
Prol06	0.0127	0.0034	0.3548	0.1009	5312 switches
Prol07	0.4926	0.0239	-0.0249	-0.0355	3613 switches
Prol08	0.0007	0.0007	0.2867	0.2124	2804 switches
Prol10	0.0600	0.0181	0.1111	0.1370	1837 switches
Prol11	0.0000	0.0000	0.4432	0.3493	4445 switches
Prol12	0.0000	0.0000	0.5520	0.4347	3877 switches
Prol19	0.0000	0.0000	0.7857	0.7185	4721 switches
Prol53	0.0000	0.0000	0.8400	0.4967	9286 switches
Prol54	0.0079	0.0037	0.4295	0.3365	3100 switches
Prol57	0.0954	0.0150	0.2010	0.0747	2647 switches

All (Fisher's method):
Chi2 : > 242.1400
Df : 30
Prob : < 4.95e-35

Pop : Cluster6

Fis estimates
----

locus	P-val	S.E.	W&C	R&H	Steps
Prol01	0.0000	0.0000	0.8033	0.6179	3179 switches

Prol02	0.0609	0.0123	0.2982	0.1329	3304 switches
Prol03	0.0000	0.0000	0.5270	0.3527	2723 switches
Prol04	0.0001	0.0001	0.4263	0.3819	4028 switches
Prol05	0.0000	0.0000	0.4089	0.3183	2791 switches
Prol06	0.0000	0.0000	0.7067	0.7086	12521 switches
Prol07	0.0460	0.0090	0.0280	-0.0110	3646 switches
Prol08	0.0000	0.0000	0.5103	0.2714	3447 switches
Prol10	0.1791	0.0249	0.0761	0.0410	1648 switches
Prol11	0.0829	0.0180	0.2174	0.0680	3764 switches
Prol12	0.0000	0.0000	0.7037	0.6271	6474 switches
Prol19	0.0000	0.0000	0.8636	0.6993	4650 switches
Prol53	0.0915	0.0204	0.2030	0.0383	2002 switches
Prol54	0.0000	0.0000	0.5281	0.3579	1561 switches
Prol57	0.0000	0.0000	0.3404	0.2669	3637 switches

All (Fisher's method):
Chi2 : > 249.6716
Df : 30
Prob : < 1.75e-36

Pop : Cluster7

Fis estimates
----

locus	P-val	S.E.	W&C	R&H	Steps
Prol01	0.0288	0.0044	0.4435	0.5229	7169 switches
Prol02	0.0025	0.0014	0.4410	0.2667	4679 switches
Prol03	0.0295	0.0079	0.2125	0.0995	3254 switches
Prol04	0.0000	0.0000	0.6117	0.5531	4332 switches
Prol05	0.0000	0.0000	0.4819	0.3000	3242 switches
Prol06	0.0129	0.0025	0.5322	0.6310	8383 switches
Prol07	0.5616	0.0293	-0.0667	-0.0437	1699 switches
Prol08	0.7827	0.0122	0.1064	0.0714	4561 switches
Prol10	0.1238	0.0180	0.0667	0.0317	1891 switches
Prol11	0.0000	0.0000	0.5115	0.2986	1871 switches
Prol12	0.0000	0.0000	0.8710	0.8700	8166 switches
Prol19	0.2807	0.0165	0.1340	0.0833	5012 switches
Prol53	0.0000	0.0000	0.6667	0.3500	5141 switches
Prol54	0.0044	0.0037	0.4175	0.4194	3041 switches
Prol57	0.0021	0.0011	0.5232	0.3940	5345 switches

All (Fisher's method):
Chi2 : > 181.5074
Df : 30
Prob : < 1.34e-23

Pop : Cluster8

Fis estimates
----

locus P-val S.E. W&C R&H Steps

----------- ------- ------- ------- ------- ------

Prol01	0.0000	0.0000	0.7581	0.6017	3364 switches
Prol02	0.0000	0.0000	0.3822	0.3013	1627 switches
Prol03	0.0000	0.0000	0.6061	0.5736	3816 switches
Prol04	0.0000	0.0000	0.5817	0.4349	3405 switches
Prol05	0.0000	0.0000	0.4493	0.3292	1284 switches
Prol06	0.0000	0.0000	0.8035	0.5881	20775 switches
Prol07	0.3523	0.0213	0.1608	0.2037	3942 switches
Prol08	0.0000	0.0000	0.3712	0.2288	6982 switches
Prol10	0.1375	0.0244	-0.0685	-0.0385	1250 switches
Prol11	0.0000	0.0000	0.6584	0.3724	3132 switches
Prol12	0.0000	0.0000	0.8061	0.8015	5116 switches
Prol19	0.0000	0.0000	0.9097	0.9938	11417 switches
Prol53	0.0000	0.0000	0.7764	0.4115	7715 switches
Prol54	0.0000	0.0000	0.4477	0.3386	3084 switches
Prol57	0.0024	0.0024	0.3097	0.2260	1771 switches

All (Fisher's method):
Chi2 : > 294.4205
Df : 30
Prob : < 2.80e-45

==

All locus, all populations

==

All (Fisher's method) :
Chi2 : > 2077.3398
Df : 240
Prob : High. sign.

===

Results from GENEPOP ORIGINAL POPULATION SAMPLES

Number of populations detected: 4
Number of loci detected: 15

Estimation of exact P-Values by the Markov chain method.

Markov chain parameters for all tests:
Dememorization: 1000
Batches: 100
Iterations per batch: 1000
Hardy Weinberg: Probability test
        ****

==
    Results by locus
==

Locus "Prol01"

POP	P-val	S.E.	Fis estimates W&C	Fis estimates R&H	Steps
Camargos	0.0023	0.0023	0.3138	0.2296	1986 switches
Itutinga_1	0.0000	0.0000	0.7079	0.6000	3236 switches
Itutinga_2	0.0000	0.0000	0.6875	0.5963	1890 switches
Funil	0.0000	0.0000	0.8400	0.8913	3725 switches

All (Fisher's method):
Chi2:   > 107.8303
Df   :   8
Prob :   < 1.06e-19

Locus "Prol02"

POP	P-val	S.E.	Fis estimates W&C	Fis estimates R&H	Steps
Camargos	0.0000	0.0000	0.3588	0.2243	1445 switches
Itutinga_1	0.0000	0.0000	0.4179	0.2707	2208 switches
Itutinga_2	0.0000	0.0000	0.4017	0.3344	2321 switches
Funil	0.0000	0.0000	0.4750	0.3198	2512 switches

All (Fisher's method):
Chi2:   > 127.5391
Df   :   8
Prob :   < 9.15e-24

```
Locus "Prol03"
-----------------------------------------
                         Fis estimates
                        ---------------
POP          P-val   S.E.    W&C     R&H     Steps
----------- ------- ------- ------- ------- ------
Camargos    0.0000  0.0000   0.6832  0.5914   1809 switches
Itutinga_1  0.0000  0.0000   0.4480  0.3247   3131 switches
Itutinga_2  0.0000  0.0000   0.6226  0.5386   2684 switches
Funil       0.0000  0.0000   0.4683  0.4023   1803 switches

All (Fisher's method):
 Chi2:     > 127.5391
 Df   :    8
 Prob :     < 9.15e-24

Locus "Prol04"
-----------------------------------------
                         Fis estimates
                        ---------------
POP          P-val   S.E.    W&C     R&H     Steps
----------- ------- ------- ------- ------- ------
Camargos    0.0000  0.0000   0.5484  0.5125   5397 switches
Itutinga_1  0.0000  0.0000   0.6697  0.6286   7359 switches
Itutinga_2  0.0000  0.0000   0.7067  0.6127   4968 switches
Funil       0.0000  0.0000   0.4507  0.3127   3547 switches

All (Fisher's method):
 Chi2:     > 127.5391
 Df   :    8
 Prob :     < 9.15e-24

Locus "Prol05"
-----------------------------------------
                         Fis estimates
                        ---------------
POP          P-val   S.E.    W&C     R&H     Steps
----------- ------- ------- ------- ------- ------
Camargos    0.0000  0.0000   0.6243  0.5577   1885 switches
Itutinga_1  0.0000  0.0000   0.3610  0.2564    850 switches (low!)
Itutinga_2  0.0000  0.0000   0.5884  0.4383   1143 switches
Funil       0.0000  0.0000   0.4272  0.3643    714 switches (low!)

All (Fisher's method):
 Chi2:     > 127.5391
 Df   :    8
 Prob :     < 9.15e-24

Locus "Prol06"
-----------------------------------------
                         Fis estimates
                        ---------------
```

```
POP          P-val   S.E.    W&C     R&H     Steps
----------- ------- ------- ------- ------- ------
Camargos    0.0000  0.0000   0.4968  0.3976   4120 switches
Itutinga_1  0.0000  0.0000   0.7684  0.8639   8679 switches
Itutinga_2  0.0000  0.0000   0.8790  0.6987   3216 switches
Funil       0.0000  0.0000   0.7456  0.6825   3408 switches

All (Fisher's method):
 Chi2:     > 127.5391
 Df   :    8
 Prob :     < 9.15e-24

Locus "Prol07"
----------------------------------------
                      Fis estimates
                      ---------------
POP          P-val   S.E.    W&C     R&H     Steps
----------- ------- ------- ------- ------- ------
Camargos    0.0191  0.0057   0.1720  0.2371   4039 switches
Itutinga_1  0.0157  0.0051  -0.0556 -0.0437   3619 switches
Itutinga_2  0.0498  0.0147   0.1220  0.1105   1521 switches
Funil       0.4959  0.0227  -0.0539 -0.0423   6736 switches

All (Fisher's method):
 Chi2:    23.6291
 Df   :    8
 Prob :    0.002644

Locus "Prol08"
----------------------------------------
                      Fis estimates
                      ---------------
POP          P-val   S.E.    W&C     R&H     Steps
----------- ------- ------- ------- ------- ------
Camargos    0.0000  0.0000   0.8909  0.9360  19278 switches
Itutinga_1  0.0000  0.0000   0.4859  0.4017  10720 switches
Itutinga_2  0.0000  0.0000   0.2819  0.1889   4871 switches
Funil       0.0000  0.0000   0.2526  0.2217   4461 switches

All (Fisher's method):
 Chi2:     > 127.5391
 Df   :    8
 Prob :     < 9.15e-24

Locus "Prol10"
----------------------------------------
                      Fis estimates
                      ---------------
POP          P-val   S.E.    W&C     R&H     Steps
----------- ------- ------- ------- ------- ------
Camargos    0.0049  0.0026   0.2254  0.2783   4671 switches
Itutinga_1  0.0000  0.0000   0.2373  0.1734   1893 switches
```

```
Itutinga_2  0.0000  0.0000  -0.0767 -0.0536   3220 switches
Funil       0.7323  0.0319  -0.0544 -0.0384   1591 switches

All (Fisher's method):
 Chi2:     > 75.0339
 Df   :    8
 Prob :     < 4.86e-13

Locus "Prol11"
----------------------------------------
                        Fis estimates
                       ---------------
POP         P-val   S.E.    W&C     R&H     Steps
----------- ------- ------- ------- ------- ------
Camargos    0.0000  0.0000   0.6198  0.5598   3051 switches
Itutinga_1  0.0000  0.0000   0.3803  0.2926   2371 switches
Itutinga_2  0.0000  0.0000   0.6995  0.6297   2094 switches
Funil       0.0000  0.0000   0.5787  0.4817   2103 switches

All (Fisher's method):
 Chi2:     > 127.5391
 Df   :    8
 Prob :     < 9.15e-24

Locus "Prol12"
----------------------------------------
                        Fis estimates
                       ---------------
POP         P-val   S.E.    W&C     R&H     Steps
----------- ------- ------- ------- ------- ------
Camargos    0.0000  0.0000   1.0000  1.0833  11722 switches
Itutinga_1  0.0000  0.0000   0.8252  0.8074   5725 switches
Itutinga_2  0.0000  0.0000   0.6335  0.4921   2649 switches
Funil       0.0000  0.0000   0.6871  0.5989   1489 switches

All (Fisher's method):
 Chi2:     > 127.5391
 Df   :    8
 Prob :     < 9.15e-24

Locus "Prol19"
----------------------------------------
                        Fis estimates
                       ---------------
POP         P-val   S.E.    W&C     R&H     Steps
----------- ------- ------- ------- ------- ------
Camargos    0.0004  0.0004   0.4430  0.4462   4223 switches
Itutinga_1  0.0000  0.0000   0.7503  0.6719   6023 switches
Itutinga_2  0.0000  0.0000   0.8599  0.8455   5548 switches
Funil       0.0000  0.0000   0.8385  0.8297   4321 switches

All (Fisher's method):
```

Chi2: > 111.5131
Df : 8
Prob : < 1.86e-20

Locus "Prol53"

Fis estimates

POP	P-val	S.E.	W&C	R&H	Steps
Camargos	0.0000	0.0000	0.5845	0.3332	3758 switches
Itutinga_1	0.0000	0.0000	0.5318	0.2256	4472 switches
Itutinga_2	0.0000	0.0000	0.7379	0.2994	2561 switches
Funil	0.0000	0.0000	0.6774	0.2606	2590 switches

All (Fisher's method):
Chi2: > 127.5391
Df : 8
Prob : < 9.15e-24

Locus "Prol54"

Fis estimates

POP	P-val	S.E.	W&C	R&H	Steps
Camargos	0.0000	0.0000	0.5676	0.5567	1529 switches
Itutinga_1	0.0000	0.0000	0.5951	0.4096	1587 switches
Itutinga_2	0.0000	0.0000	0.6898	0.5938	2034 switches
Funil	0.0052	0.0031	0.2244	0.1611	3468 switches

All (Fisher's method):
Chi2: > 106.1725
Df : 8
Prob : < 2.33e-19

Locus "Prol57"

Fis estimates

POP	P-val	S.E.	W&C	R&H	Steps
Camargos	0.0000	0.0000	0.5493	0.4477	3917 switches
Itutinga_1	0.0000	0.0000	0.4916	0.4760	4206 switches
Itutinga_2	0.0210	0.0126	0.1420	0.0798	1207 switches
Funil	0.0000	0.0000	0.2504	0.1849	1305 switches

All (Fisher's method):
Chi2: > 103.3770
Df : 8
Prob : < 8.70e-19

```
========================================
    Results by population
========================================


Pop : Camargos
-----------------------------------------
                        Fis estimates
                       ---------------
locus       P-val   S.E.    W&C     R&H     Steps
----------- ------- ------- ------- ------- ------
Prol01      0.0023  0.0023   0.3138  0.2296   1986 switches
Prol02      0.0000  0.0000   0.3588  0.2243   1445 switches
Prol03      0.0000  0.0000   0.6832  0.5914   1809 switches
Prol04      0.0000  0.0000   0.5484  0.5125   5397 switches
Prol05      0.0000  0.0000   0.6243  0.5577   1885 switches
Prol06      0.0000  0.0000   0.4968  0.3976   4120 switches
Prol07      0.0191  0.0057   0.1720  0.2371   4039 switches
Prol08      0.0000  0.0000   0.8909  0.9360  19278 switches
Prol10      0.0049  0.0026   0.2254  0.2783   4671 switches
Prol11      0.0000  0.0000   0.6198  0.5598   3051 switches
Prol12      0.0000  0.0000   1.0000  1.0833  11722 switches
Prol19      0.0004  0.0004   0.4430  0.4462   4223 switches
Prol53      0.0000  0.0000   0.5845  0.3332   3758 switches
Prol54      0.0000  0.0000   0.5676  0.5567   1529 switches
Prol57      0.0000  0.0000   0.5493  0.4477   3917 switches

All (Fisher's method):
 Chi2 :     > 299.8722
 Df   :    30
 Prob :     < 0.00e+00

Pop : Itutinga_1
-----------------------------------------
                        Fis estimates
                       ---------------
locus       P-val   S.E.    W&C     R&H     Steps
----------- ------- ------- ------- ------- ------
Prol01      0.0000  0.0000   0.7079  0.6000   3236 switches
Prol02      0.0000  0.0000   0.4179  0.2707   2208 switches
Prol03      0.0000  0.0000   0.4480  0.3247   3131 switches
Prol04      0.0000  0.0000   0.6697  0.6286   7359 switches
Prol05      0.0000  0.0000   0.3610  0.2564    850 switches (low!)
Prol06      0.0000  0.0000   0.7684  0.8639   8679 switches
Prol07      0.0157  0.0051  -0.0556 -0.0437   3619 switches
Prol08      0.0000  0.0000   0.4859  0.4017  10720 switches
Prol10      0.0000  0.0000   0.2373  0.1734   1893 switches
Prol11      0.0000  0.0000   0.3803  0.2926   2371 switches
Prol12      0.0000  0.0000   0.8252  0.8074   5725 switches
Prol19      0.0000  0.0000   0.7503  0.6719   6023 switches
Prol53      0.0000  0.0000   0.5318  0.2256   4472 switches
Prol54      0.0000  0.0000   0.5951  0.4096   1587 switches
```

```
Prol57      0.0000  0.0000   0.4916  0.4760   4206 switches

All (Fisher's method):
 Chi2 :     > 330.6752
 Df   :    30
 Prob :     < 0.00e+00

Pop : Itutinga_2
-----------------------------------------
                        Fis estimates
                       ---------------
locus       P-val   S.E.    W&C     R&H     Steps
----------- ------- ------- ------- ------- ------
Prol01      0.0000  0.0000   0.6875  0.5963   1890 switches
Prol02      0.0000  0.0000   0.4017  0.3344   2321 switches
Prol03      0.0000  0.0000   0.6226  0.5386   2684 switches
Prol04      0.0000  0.0000   0.7067  0.6127   4968 switches
Prol05      0.0000  0.0000   0.5884  0.4383   1143 switches
Prol06      0.0000  0.0000   0.8790  0.6987   3216 switches
Prol07      0.0498  0.0147   0.1220  0.1105   1521 switches
Prol08      0.0000  0.0000   0.2819  0.1889   4871 switches
Prol10      0.0000  0.0000  -0.0767 -0.0536   3220 switches
Prol11      0.0000  0.0000   0.6995  0.6297   2094 switches
Prol12      0.0000  0.0000   0.6335  0.4921   2649 switches
Prol19      0.0000  0.0000   0.8599  0.8455   5548 switches
Prol53      0.0000  0.0000   0.7379  0.2994   2561 switches
Prol54      0.0000  0.0000   0.6898  0.5938   2034 switches
Prol57      0.0210  0.0126   0.1420  0.0798   1207 switches

All (Fisher's method):
 Chi2 :     > 313.0598
 Df   :    30
 Prob :     < 0.00e+00

Pop : Funil
-----------------------------------------
                        Fis estimates
                       ---------------
locus       P-val   S.E.    W&C     R&H     Steps
----------- ------- ------- ------- ------- ------
Prol01      0.0000  0.0000   0.8400  0.8913   3725 switches
Prol02      0.0000  0.0000   0.4750  0.3198   2512 switches
Prol03      0.0000  0.0000   0.4683  0.4023   1803 switches
Prol04      0.0000  0.0000   0.4507  0.3127   3547 switches
Prol05      0.0000  0.0000   0.4272  0.3643    714 switches (low!)
Prol06      0.0000  0.0000   0.7456  0.6825   3408 switches
Prol07      0.4959  0.0227  -0.0539 -0.0423   6736 switches
Prol08      0.0000  0.0000   0.2526  0.2217   4461 switches
Prol10      0.7323  0.0319  -0.0544 -0.0384   1591 switches
Prol11      0.0000  0.0000   0.5787  0.4817   2103 switches
Prol12      0.0000  0.0000   0.6871  0.5989   1489 switches
Prol19      0.0000  0.0000   0.8385  0.8297   4321 switches
```

Prol53	0.0000	0.0000	0.6774	0.2606	2590 switches
Prol54	0.0052	0.0031	0.2244	0.1611	3468 switches
Prol57	0.0000	0.0000	0.2504	0.1849	1305 switches

All (Fisher's method):
Chi2 : > 288.8544
Df : 30
Prob : < 4.06e-44

==

All locus, all populations

==

All (Fisher's method) :
Chi2 : > 1232.4615
Df : 120
Prob : High. Sign.

==
